# From Preparedness to Documented Operational Readiness: Mapping Public Evidence Across the EU/EEA Health-Security Architecture

**DOI:** 10.3390/healthcare14203529

**Published:** 2026-10-20

**Authors:** Krzysztof Goniewicz, Henk van der Wal, Markus Ries, Amir Khorram-Manesh

**Affiliations:** 1 Department of Security Studies, Polish Air Force University, 08-521 Dęblin, Poland; 2 Civil-Military Centre of Expertise for Trauma Care, Central Military Hospital, Ministry of Defence, 3584 EZ Utrecht, The Netherlands; h.vanderwal@umcutrecht.nl; 3 Civil-Military Centre of Expertise for Trauma Care, Traumacenter, University Medical Center Utrecht, 3584 CX Utrecht, The Netherlands; 4 Crisis and Disaster Management Unit, Heidelberg University Hospital, Heidelberg University, Im Neuenheimer Feld 430, 69120 Heidelberg, Germany; markus.ries@uni-heidelberg.de; 5 Center for Virtual Patients, Medical Faculty, University of Heidelberg, 69120 Heidelberg, Germany; 6 Pediatric Neurology and Metabolic Medicine, Center for Pediatrics and Adolescent Medicine, Heidelberg University Hospital, 69120 Heidelberg, Germany; 7 Rhine-Neckar Public Health Service, 69115 Heidelberg, Germany; 8 Department of Surgery, Institute of Clinical Sciences, Sahlgrenska Academy, Gothenburg University, 405 30 Gothenburg, Sweden; 9 Disaster Medicine Center, University of Gothenburg, 405 30 Gothenburg, Sweden; 10 Gothenburg Emergency Medicine Research Group (GEMREG), Sahlgrenska Academy, Gothenburg University, 405 30 Gothenburg, Sweden

**Keywords:** documented operational readiness, health emergency preparedness, health security, European Union, documentary analysis, health-system resilience

## Abstract

**Highlights:**

**What are the main findings?**

- Public sources describe EU/EEA health-security policies and established mechanisms more often than completed exercises or use during real events.
- Among 175 observations included in the quantitative analysis, 62 contained explicitly documented gaps. None met this study’s strict criteria for a hybrid, compound, or cascading configuration.

**What are the implications of the main findings?**

- Public-document assessments should distinguish reported implementation gaps from missing evidence and avoid treating either as a direct measure of operational performance.
- The shared codebook and coding matrix provide a reproducible basis for future documentary assessments and help identify where exercise and after-action evidence are needed.

**Abstract:**

**Background/Objectives**: Post-pandemic reforms have expanded the EU/EEA health-security architecture across surveillance, crisis coordination, medical countermeasures, workforce mobilisation, civil protection and cybersecurity. Preparedness structures do not themselves establish operational performance. We mapped publicly available documentary evidence of operationalisation across eight health-readiness domains. **Methods**: We applied structured qualitative documentary analysis and a capability-based Framework Method to 35 primary public sources dated or accessed between 1 January 2024 and 17 September 2026. We extracted 190 source-linked observations. Frequency estimates included 175 observations from 34 sources after excluding 15 potentially overlapping rows from an interim cross-country PHEPA synthesis. Evidence was coded using nonexclusive policy (P), mechanism (M), operational functionality (O), completed exercise (E) and real-world use (R) attributes, without deriving a readiness score. **Results**: Policy commitments and established mechanisms were documented more often than completed exercise testing or real-world use. Explicitly documented gaps occurred in 62 of 175 observations (35.4%), concerning formalisation and activation, interoperability and situational visibility, scalability and sustainability, exercise and event evidence, learning and corrective-action closure, and cross-sector or cross-border integration. Gap frequencies differed by source type, particularly between PHEPA reports and other documents, and do not indicate comparative readiness. Most observations did not specify hazard interaction; none met the revised strict thresholds for a hybrid campaign, compound crisis or cascading disruption. **Conclusions**: This study provides a traceable map of public evidence linking preparedness structures to reported operationalisation. Its findings identify documentary patterns and implementation challenges, not direct measures of capability, effectiveness or performance in complex crises.

## 1. Introduction

The COVID-19 pandemic exposed persistent vulnerabilities in health-system surge capacity, supply chains, surveillance, cross-border coordination and continuity of essential services, while simultaneously accelerating institutional reform in European health governance. In the years that followed, the European Union (EU) moved beyond the emergency arrangements developed during the pandemic toward a more permanent health-security architecture. Regulation (EU) 2022/2371 strengthened the framework for prevention, preparedness and response to serious cross-border threats to health, including preparedness planning, epidemiological surveillance, early warning, risk assessment and coordinated response [1]. The establishment and subsequent development of the Health Emergency Preparedness and Response Authority (HERA) added dedicated capabilities for health-threat intelligence, medical-countermeasure development, manufacturing, procurement and deployment [2]. These reforms have progressively embedded health emergencies within a broader European framework of resilience and crisis management rather than treating them solely as episodic public-health events.

This evolution has continued as the European risk environment has broadened. The European Preparedness Union Strategy, adopted in March 2025, explicitly frames preparedness through integrated all-hazards, whole-of-government and whole-of-society approaches and links crisis readiness to health emergencies, critical infrastructure, cybersecurity, information manipulation, civil protection and civil–military cooperation [3]. The 2025 Medical Countermeasures Strategy similarly extends health preparedness across an end-to-end chain encompassing threat assessment, research and development, scalable manufacturing, stockpiling, procurement and rapid deployment [4]. At the same time, EU policy increasingly recognises that crises may originate from natural, technological or deliberately induced threats and may affect several sectors simultaneously. Hybrid threats, in EU terminology, involve coordinated harmful activities undertaken with malign intent through multiple and potentially combined instruments, including cyberattacks, information manipulation, economic coercion and other disruptive means [5].

Our earlier policy analysis [6] characterised post-COVID EU health-governance reform through 2020–2025 legislation and strategies, focusing on institutional consolidation, digital governance, medical countermeasures and the health–security nexus. Here, the question moves from how EU governance changed to what public evidence supports each specified health-security capability. We code source-located document–capability observations across eight domains for formal commitments, mechanisms, reported operation, completed exercises and real-world use, adding national implementation assessments and explicitly documented gaps. This evidence map makes particular claims inspectable; it cannot show that reforms improved emergency performance.

This distinction is captured by the emerging concept of operational readiness. English et al. describe operational readiness as occupying the space between longer-term preparedness and emergency response, involving the immediate capabilities and actions required to respond rapidly to an imminent or emerging public-health threat [7]. Their review also demonstrated that operational readiness remains less consistently conceptualised than either preparedness or response and that readiness-related activities are often embedded within broader preparedness frameworks rather than identified explicitly [7]. The World Health Organization (WHO) similarly places operational readiness at the interface between established capacities and effective response. Its Health Emergency Preparedness, Response and Resilience (HEPR) architecture identifies five interconnected systems, collaborative surveillance, community protection, safe and scalable care, access to countermeasures and emergency coordination, whose functioning depends on underlying capacity and operational readiness [8]. More recent WHO guidance has continued this emphasis on translating preparedness structures into coordinated multi-hazard response functions rather than maintaining preparedness as a predominantly planning-oriented activity [9].

The distinction becomes particularly important when health systems are confronted with hybrid, compound or cascading crises. A hybrid campaign is defined principally by deliberate intent and the coordinated use of multiple instruments [10]. The disaster-risk literature distinguishes compound, interacting, interconnected and cascading risks and cautions against treating these terms as interchangeable [11,12]. In the literature, compound risk concerns the interaction of multiple drivers, hazards or processes, whereas cascading risk concerns the propagation of consequences from an initiating event through interconnected systems. More generally, formal measures of preparedness should not be taken as direct evidence of response performance: an exploratory analysis of 177 countries found that pandemic-preparedness indices were not meaningfully associated with standardised COVID-19 infection rates or infection-fatality ratios [13]. For this documentary study, we applied deliberately conservative coding rules: a compound crisis required an explicit description of concurrent interacting hazards or pressures affecting a shared capability, population or service, and cascading disruption required an explicit description of an initiating event producing secondary failures in dependent systems that affected health-system functioning. These thresholds classify the framing contained in public documents; they are not intended as exhaustive definitions of all compound or cascading crises. Thus, an infectious-disease outbreak occurring alongside energy disruption, workforce shortages and supply-chain failure was classified as compound only when the interaction was explicit and as cascading only when the document described a causal propagation chain; otherwise, it remained a multi-hazard context. Conversely, coordinated cyberattacks, information manipulation and deliberate disruption of health infrastructure may constitute a hybrid threat even if the initial direct health impact is limited [14]. This distinction is increasingly relevant because modern healthcare depends on tightly coupled digital, energy, transport, manufacturing, laboratory and logistics systems. The EU Preparedness Union Strategy itself reflects this broader understanding of crisis interaction by linking preparedness across sectors and by emphasising comprehensive risk assessment, critical societal functions and regular exercises [3].

Despite this expansion of European preparedness policy, publicly available evidence that formal arrangements have been operationalised remains fragmented. Strategies and legislation can establish mandates; programmes can create mechanisms and resources; exercises can test selected components; and actual emergencies can provide evidence of deployment. These represent different forms of evidence and should not be treated as equivalent indicators of readiness. The distinction is particularly important when assessing newer capabilities related to cyber resilience, civil–military coordination, information integrity or deliberate threats, for which public documentation of real-world activation may be inherently more limited. Conversely, established mechanisms such as medical evacuation, deployable public-health expertise or medical-countermeasure procurement may generate observable operational evidence without demonstrating the readiness of the entire system in which they operate.

The introduction of the European Centre for Disease Prevention and Control (ECDC) Public Health Emergency Preparedness Assessments (PHEPAs) provides an important additional evidence layer. Conducted under the framework established by Regulation (EU) 2022/2371, PHEPAs examine implementation of national prevention, preparedness and response capacities rather than only the formal existence of plans [1]. The first assessment cycle began in 2024, and the 2026 interim synthesis of the first 15 assessments identified both recurrent strengths and common implementation challenges across surveillance, laboratories, health-emergency management and related preparedness capacities [15]. ECDC explicitly cautions that these findings are not a comprehensive comparative assessment of EU/EEA preparedness, but they provide an opportunity to examine the interface between European-level architecture and national-level implementation.

Complementing this top-down perspective, a local lessons-learned study of civil–military pandemic response in Heidelberg/Rhine-Neckar identified practical preparedness themes, including local alignment, community networks and relationships, clear leadership roles and responsibilities, timely transition into disaster mode, personnel reserves, frequent all-hands exercises and functioning as a learning organisation [16]. Although derived from a single regional case, these findings illustrate how operational readiness is also shaped by local relationships, role clarity and learning processes that may not be fully visible in supranational policy architecture.

Taken together, the current policy landscape creates a need to distinguish the existence of preparedness structures from the types of publicly available evidence supporting their operationalisation. The existing literature has conceptualised operational readiness, and international organisations have developed increasingly detailed preparedness frameworks, but there has been limited systematic examination of operationalisation across the contemporary EU/European Economic Area (EU/EEA) health-security architecture as a whole. It remains unclear how consistently formal commitments are supported by publicly documented evidence of established mechanisms, operational functionality, completed exercise-based testing or real-world use across the EU/EEA health-security architecture. It is also unclear how the included documents frame deliberate, hybrid, compound and cascading disruptions.

Accordingly, this study examines publicly available documentary evidence of operationalisation within the emerging EU/EEA health-security architecture. It addresses three questions: (1) Which operational health-readiness capabilities are represented in the current EU/EEA preparedness architecture? (2) What documentary evidence exists beyond formal policy commitments for established mechanisms, operational functionality, exercise-based testing and real-world use? (3) How are deliberate, hybrid, compound and cascading disruptions framed in the included public documents? The third question is descriptive and does not constitute a direct assessment of readiness for these specific crisis configurations. Rather than generating a composite readiness score or ranking national systems, this study distinguishes different forms of evidence of operationalisation and examines where preparedness architecture is supported by publicly documented evidence of operational functionality, scalability, exercise-based testing and adaptive learning.

## 2. Methods

### 2.1. Study Design and Analytical Scope

This study used a structured qualitative policy analysis combined with a capability-based Framework Method to examine publicly available documentary evidence of operationalisation within the emerging European Union/European Economic Area (EU/EEA) health-security architecture. Document analysis was selected because this study sought to examine the relationship between formal policy architecture, implementation mechanisms, and documented evidence of operationalisation rather than to estimate intervention effects or clinical outcomes [17,18].

Operational readiness was conceptualised as the intermediate state between longer-term preparedness and emergency response, in which existing plans, resources, structures, and capacities can be rapidly activated, mobilised, scaled, and deployed in response to an emerging or imminent threat. This conceptualisation was informed by the rapid scoping review of operational readiness by English et al. and by the WHO HEPR architecture [7,8]. The WHO identifies five interconnected health-emergency systems, collaborative surveillance, community protection, safe and scalable care, access to countermeasures, and emergency coordination, whose effectiveness depends on operational readiness and underlying capacities. The 2026 WHO Framework for Health Emergency Preparedness and Response Capabilities for National Public Health Agencies was additionally used to refine the analytical domains and distinguish technical capabilities from cross-cutting enabling functions [19].

Throughout this study, “documented operational readiness” refers to the type and extent of publicly available evidence supporting a claim that a capability has been operationalised. It is not a direct measure of the underlying capability, its effectiveness, or the completeness of arrangements that may remain non-public. Accordingly, absence of public evidence was not interpreted as evidence that a capability was absent.

The primary analytical period was 1 January 2024 to 17 September 2026. Earlier documents were retained only when they constituted an essential legal or conceptual foundation for the contemporary architecture, including Regulation (EU) 2022/2371 on serious cross-border threats to health and relevant WHO preparedness frameworks. Regulation (EU) 2022/2371 was treated as a foundational legal instrument because it establishes Union-level arrangements for preparedness planning, surveillance, early warning, risk assessment, crisis communication, medical countermeasures, and continuity of healthcare services and provides the legal basis for periodic ECDC assessment of national prevention, preparedness and response planning [1].

### 2.2. Documentary Corpus and Source Hierarchy

Documents were identified from publicly available sources maintained by EU institutions, bodies, agencies and repositories, including the European Commission, particularly the Directorate-General for Health and Food Safety and HERA; ECDC; the European Medicines Agency; the Directorate-General for European Civil Protection and Humanitarian Aid Operations and the Union Civil Protection Mechanism; the European Union Agency for Cybersecurity (ENISA); the European External Action Service; the Council of the European Union; and EUR-Lex.

Eligible sources included legislation, Commission communications, strategies, preparedness and response plans, technical guidance, implementation reports, country assessments, operational reports, exercise reports, after-action material, deployment reports, and other official documents containing substantive evidence relevant to one or more predefined readiness capabilities.

Particular attention was given to ECDC PHEPAs. Under Article 8 of Regulation (EU) 2022/2371 and the associated ECDC assessment process, PHEPAs are conducted across EU/EEA countries on a three-year cycle to assess implementation of national prevention, preparedness and response planning. PHEPA reports were, therefore, treated as the principal national-level implementation evidence rather than simply as additional policy documents [1,20].

WHO documents were used as external conceptual and technical benchmarks rather than as evidence of EU/EEA implementation. The 2026 NATO Resilience Baseline Requirements were added after closure of the primary search as an external contextual benchmark for civil–military, mass-casualty and disruptive-health-crisis dimensions [21]. They were not included in the primary analytical corpus and did not determine eligibility, source selection, observation coding or reported results.

A complete inventory of the primary analytic sources, supporting/contextual sources, and foundational or benchmark documents, including their provenance and analytical role, is provided in Supplementary Material S1.

### 2.3. Search Strategy and Document Identification

Documents were identified through a purposive, structured search of institutional websites and repositories, supplemented by targeted domain-restricted web searches. Search concepts covered operational readiness, health emergency preparedness, health security, surge capacity, continuity of essential health services, medical countermeasures, strategic stockpiling, emergency coordination, civil–military health preparedness, hybrid threats, deliberate events, cybersecurity, critical infrastructure, compound crises, cascading disruption, exercises, deployment, and emergency logistics.

The core concepts were combined iteratively according to the mandate, structure, and terminology of each institution rather than applying a single identical search string across all repositories. Examples included combinations of “operational readiness”, “health emergency preparedness”, “surge capacity”, “medical countermeasures”, stockpil*, “civil-military”, “hybrid threat*”, cyber*, “continuity of essential health services”, exercise*, and deployment with terms related to health, hospitals, public health, the European Union, and the EEA.

Supplementary Material S2 lists the repositories, representative search combinations, eligibility rules and retained-source disposition; Supplementary Material S1 inventories all 45 retained documents (35 primary analytical, 3 supporting/contextual and 7 foundational/benchmark). The iterative search closed on 17 September 2026. No formal saturation criterion was specified or assessed. The contemporaneous search record did not preserve the dates of individual repository queries, the number of candidates identified or screened, or record-level exclusion decisions. These counts and the distribution of exclusion reasons cannot be reconstructed reliably. Section 2.4 states the exclusion criteria. The disposition table, therefore, reports retained sources without a PRISMA-like screening flow.

### 2.4. Eligibility Criteria

Documents were eligible for the primary analysis when they concerned the EU/EEA or one or more EU/EEA countries; addressed at least one predefined operational-readiness domain; and contained substantive information on a policy commitment, established mechanism or resource, activation procedure, mobilisation or deployment capability, completed exercise, real-world activation, implementation status, operational dependency, or explicitly documented operational gap.

Documents published between 1 January 2024 and 17 September 2026 were eligible for inclusion in the primary analytical corpus. The start date was selected to coincide with the launch of the first ECDC Public Health Emergency Preparedness Assessment cycle (2024–2026), marking the beginning of systematic implementation-focused assessment within the reformed EU health-security architecture [15,20].

Earlier documents were retained only when they were necessary to establish the legal or conceptual basis of the contemporary preparedness architecture and were classified separately as foundational or benchmark sources rather than as primary analytic evidence.

Duplicates, superseded versions where a final version was available, documents unrelated to health emergency readiness, purely routine health-system material, and commentary without substantive evidence were excluded. Speeches, promotional material, and press releases were not normally treated as primary evidence when an underlying legal, strategic, technical, implementation, or operational document was available. Official event or deployment notices were retained when they constituted the authoritative public record of a completed exercise or real-world activation.

### 2.5. Development of the Operational Readiness Framework

An a priori analytical framework was developed by cross-mapping the WHO HEPR architecture, the 2026 WHO Framework for Health Emergency Preparedness and Response Capabilities for National Public Health Agencies, ECDC PHEPA capacities, the EU Preparedness Union Strategy, and external health-security and resilience benchmarks [3,8,19,20,21]. WHO’s 2026 framework distinguishes three foundational capabilities, legal authority, evidence generation and use for policy, and secure and flexible financing, from technical capabilities covering coordination, emergency management, workforce, surveillance and intelligence, laboratory systems, risk communication and community engagement, public health and social measures, clinical care, and countermeasure research and deployment [19].

The resulting framework comprised eight operational domains presented in Table 1.

Legal authority, flexible emergency financing, and evidence-to-policy mechanisms were coded as cross-cutting enablers rather than separate operational domains. Hybrid, compound and cascading crisis types were recorded as documentary threat framing only when the source met the definitions in Supplementary Material S3; they do not imply completed scenario testing.

Detailed operational definitions, indicator-level coding rules, evidence-of-operationalisation criteria, threat-context classifications, gap coding, and ambiguity-resolution rules are provided in Supplementary Material S3.

The EU Preparedness Union Strategy provided an additional policy-level cross-check because it explicitly adopts an integrated all-hazards, whole-of-government, and whole-of-society approach and includes foresight, crisis coordination, stockpiling, public–private arrangements, civil–military cooperation, exercises, and protection against information manipulation.

### 2.6. Evidence-of-Operationalisation Coding

For every eligible document–capability observation, evidence was coded using five non-mutually exclusive categories:

1. P (Policy) indicated a formal strategy, plan, legal requirement, or explicit policy commitment.
2. M (Mechanism) indicated that a named structure, resource, procedure, stockpile, workforce arrangement, coordination mechanism, or other implementation instrument had been established.
3. O (Operational) required explicit evidence that the capability could be activated, mobilised, deployed, redistributed, or scaled in an emergency. Describing a mechanism as “operational” without information demonstrating activation or deployment functionality was not sufficient on its own.
4. E (Exercised) required evidence that the specified capability had been tested in a completed simulation, tabletop, functional, full-scale or comparable exercise. E records completion of testing, not adequate performance, achievement of exercise objectives or likely effectiveness in a real emergency. Explicitly reported exercise shortcomings were recorded separately as documented gaps.
5. R (Real-world use) required evidence of activation or deployment during an actual emergency, crisis, incident, or operational response.

The five codes are parallel evidence attributes. They are non-mutually exclusive and can be assigned in combination to one document–capability observation. Co-occurrence of codes records the different kinds of public evidence available; it does not indicate development along a scale, a ranked maturity level, or a mandatory sequence. Thus, an observation may be coded P+M+O+E+R, but any code may also occur without the others. The categories were not converted into a numerical score of documented operational readiness.

The original codebook included NR (‘not publicly reported’). Because the unit of analysis was an evidence-bearing document–capability observation, absence of public evidence could not be classified validly within the observation matrix and was, therefore, not used as an evidence code in the revised analysis. Each gap-only observation is marked ‘Documented gap only’ in a separate observation-role field.

### 2.7. Threat-Context Classification

Threat characteristics were coded independently along two dimensions. Threat-context variables were coded as descriptive characteristics of documentary framing and independently of the P/M/O/E/R evidence classification. They were used to describe the contexts explicitly represented in the corpus, not to derive configuration-specific estimates of documented operational readiness.

The first captured intent: natural/accidental, deliberate, hybrid campaign or unclear. Deliberate intent was assigned only where a deliberately caused event or harmful action was stated, and natural/accidental where a non-deliberate hazard or event was identified. Generic or mixed-intent capability descriptions were coded unclear. A hybrid campaign required coordinated harmful activities undertaken with malign intent through multiple combined instruments [10].

The second captured interaction structure: single hazard, multi-hazard, not specified/not applicable, compound crisis or cascading disruption [11,12]. Coding referred to the supporting observation and its immediate context, not automatically to the broad remit of the whole source. A single hazard was one identified hazard class or one-hazard event, even where several sectors, functions or pathogens within that class were involved. Multi-hazard required two or more distinct hazard classes or an explicit all-hazards or multi-hazard scope tied to the observation. Generic capability descriptions without an identifiable hazard configuration were coded not specified/not applicable. Compound crisis required an explicit interaction of two or more concurrent hazards or pressures affecting the same function, population or service; cascading disruption required an initiating event causing secondary failures in dependent systems that affected health-system functioning. General references to complexity, interdependence or continuity did not meet these stricter thresholds.

Artificial intelligence was coded as a cross-cutting modifier rather than an independent readiness domain using four categories: none, capability-enabling, threat-amplifying, or both. This allowed AI-supported surveillance, forecasting, logistics, or decision support to be distinguished from AI-enabled cyber or information threats.

Detailed threat-context definitions and coding rules are provided in Supplementary Material S3.

### 2.8. Data Extraction and Analytical Variables

The unit of extraction was a source-linked observation of a specified capability or explicitly reported limitation. A source contributed separate rows when its evidence independently supported distinct capabilities or separately stated gaps; multiple P/M/O/E/R attributes and a documented gap could coexist in one row without creating additional observations. One context-only threat-framing reference had no domain assignment. Each row has a unique Observation ID, Source ID, supporting passage locator and observation role in Supplementary Material S4.

For each observation, the extraction matrix recorded the document identifier, institution or source, publication date, geographic level, exact page, section or other source location, supporting evidence, readiness domain and indicator, observation role, evidence-of-operationalisation code(s), threat intent, hazard-interaction type, governance level, cross-border scalability, civil–military component, AI role, cross-cutting enabler(s), critical dependencies, gap status, documented gap or corrective recommendation, and separate threat-framing and coding-decision notes where applicable.

Dependencies were coded inductively when they materially enabled or constrained operational readiness and included factors, such as workforce availability, digital infrastructure and telecommunications, energy and utilities, transport and logistics, laboratory capacity, manufacturing and procurement, supply chains, financing, and inter-organisational or cross-sector interoperability.

No observation was created without a supporting source passage. A documented gap was counted once per included row marked “Documented gap”, irrespective of co-occurring evidence codes or the number of limitations mentioned in its passage. Explicit limitations without a positive P/M/O/E/R attribute were retained as gap-only rows. Missing O, E or R evidence did not itself create a documented gap; “No gap reported” indicates only that the cited passage did not explicitly report one. For mechanism counts, each gap row received one primary recurrent assignment.

### 2.9. Screening and Coding Procedure

Document identification and eligibility screening followed the stated institutional search and inclusion rules; the search closed on 17 September 2026. Before full extraction, the capability framework was pilot-tested on heterogeneous EU-level, national, cyber, countermeasure, exercise and deployment documents. The core domains and P/M/O/E/R evidence categories were finalised after pilot testing on 18 September 2026 and used for the original extraction. Version 1.1 (8 October 2026) documented later, non-preregistered clarification of hybrid, compound and cascading thresholds and the treatment of NR. Version 1.2 (9 October) clarified that E denotes completed testing rather than adequate performance and renamed the exercise and event evidence gap mechanism; no evidence code or numerical result changed between Versions 1.1 and 1.2. Version 1.3 (12 October) records a retrospective audit of source identity and the intent and interaction fields for all 190 observations. Two substantive, separately dated webpages were given their own IDs, and one M code for proposed cyber services was corrected. Version 1.4 (15 October) records a targeted source-to-code audit of laboratory and surveillance, cyber, and medical-evacuation claims. Two O codes and one R code unsupported by their specific source passages were removed; associated counts were recalculated. Version 1.5 (15 October) makes the observation accounting and whole-source P01 exclusion explicit; it changes neither row codes nor numerical results. Version 1.6 (15 October) aligns the codebook’s allowed interaction values with the matrix, documents a rule-consistency check and adds row-level reasons for multi-hazard assignments and boundary cases; it does not change any classification or frequency. These later decisions were not part of the original framework or preregistered. The original screening and primary coding were undertaken by one researcher; there was no independent duplicate screening or coding. Each observation retains a source URL and page or section locator in Supplementary Material S4.

The final coding framework and ambiguity-resolution rules are provided in Supplementary Material S3, while the complete extraction matrix linking each document–capability observation to its source location and coded variables is provided in Supplementary Material S4. Across the analysis, absence of a code or document–capability observation was interpreted as absence of explicitly identified public evidence in the corpus, not as evidence that the underlying capability was absent; category and documented-gap counts were treated as descriptive features of the documentary corpus, not as comparative measures of readiness, performance or national capacity.

### 2.10. Framework Analysis and Synthesis

Data were analysed using a deductive–inductive Framework Method [18]. In the deductive phase, observations were charted against the eight predefined operational-readiness domains and the P/M/O/E/R evidence categories. Framework matrices were then constructed to compare capability × operationalisation evidence × threat context × governance level × cross-border scalability × dependency × gap status.

The inductive phase identified recurrent bottlenecks, enabling conditions, cross-sector dependencies, implementation problems, and vulnerabilities not fully captured by the initial framework. Particular attention was given to differences between formal preparedness architecture and evidence of activation, scalability, exercise testing, real-world use, and subsequent learning or adaptation.

Analysis was performed at two interconnected levels. The first examined EU-level architecture and capabilities, including coordination, medical countermeasures, stockpiling, civil protection, cyber preparedness, and deployable mechanisms. The second examined national implementation evidence, principally through ECDC PHEPA documentation.

PHEPA findings were not used to construct country rankings. Because the depth of assessment was not identical across all capacities and countries, absence of a capability from a country report was not interpreted as national absence of that capability. Cross-country synthesis was restricted to recurrent patterns explicitly supported by comparable assessment material. ECDC itself cautions that the first-cycle interim synthesis of 15 assessments does not constitute a comprehensive overview of all prevention, preparedness and response capacities across the EU/EEA [15].

No aggregate measure of documented operational readiness was calculated. The analysis instead distinguished areas where operationalisation was supported by policy, mechanism, operational, exercise, or real-world evidence and areas where official sources documented implementation limitations or where adequate public evidence of operationalisation was not identified. Descriptive counts of sources and document–capability observations were used to characterise the distribution of evidence across domains and analytical categories and were not interpreted as comparative measures of readiness or performance.

As a source dependence sensitivity analysis, observation-level frequencies were calculated from individual country PHEPA reports and non-PHEPA sources. The cross-country PHEPA interim synthesis was retained for contextual interpretation but excluded in its entirety from numerical estimates because it aggregates assessments also represented by individual country reports in the corpus; its 15 rows were not matched one by one to country-report rows. We additionally conducted leave-one-country analyses excluding Cyprus alone and Cyprus and Liechtenstein together, because these reports contributed relatively large numbers of coded observations. These analyses assessed whether the descriptive distribution of evidence categories and documented gaps was sensitive to individual source concentration. No statistical testing or country-level comparison was performed.

### 2.11. Methodological Rigour

Methodological rigour was supported through an externally anchored capability framework, explicit eligibility criteria, pilot testing of core codes, source provenance and locators, an observation-level audit trail, and a distinction between reported gaps and limited public evidence. The chronology and retrospective decisions are documented in Versions 1.1–1.6 of Supplementary Material S3. These procedures make decisions inspectable but do not establish inter-rater reliability; no independent duplicate screening or coding was undertaken.

Evidence was interpreted according to source provenance and evidentiary function. Binding legislation and primary institutional documents were used to establish formal mandates and policy architecture; implementation and operational reports were preferred for claims about operationalisation; completed exercise documentation was required for assignment of E codes; and documented real-world activation or deployment was required for R codes. The peer-reviewed literature was used selectively for conceptual triangulation and interpretation and was not used to override primary institutional evidence concerning the existence or operational status of EU/EEA mechanisms or capabilities.

Coding decisions were constrained by predefined assignment and exclusion rules. Ambiguous cases were resolved conservatively, with higher levels of operationalisation assigned only where the documentary evidence met the relevant codebook threshold. Absence of publicly available evidence was not interpreted as evidence of capability absence or failure.

### 2.12. Ethics

This study analysed publicly available policy, legal, technical, and operational documents and did not involve human participants, identifiable personal information, or individual-level health data. Formal ethics review and informed consent were, therefore, not required.

## 3. Results

### 3.1. Characteristics of the Documentary Corpus

The final primary analytical corpus comprised 35 sources dated within the study period from 1 January 2024 to 17 September 2026. Thus 22 represented EU-level policy, implementation, operational, exercise or deployment evidence, while 13 provided ECDC preparedness-assessment evidence, comprising 12 publicly available country-level PHEPA reports and the interim synthesis of the first 15 assessments [15]. Further, 1 source was dated 2024, 17 were dated 2025, and 17 were dated 2026. Two separately dated, substantively coded webpages previously grouped under EU11 and EU15 were assigned distinct source IDs (EU22 and EU23). Table 2 summarises the corpus; Supplementary Material S1 provides the complete inventory.

The corpus deliberately combined evidence on formal preparedness architecture with documentation of implementation and operationalisation. EU-level sources included strategic and regulatory initiatives, health-crisis planning instruments, HERA work programmes, medical-countermeasure and stockpiling mechanisms, cybersecurity initiatives, deployable workforce arrangements, civil-protection capacities, and completed simulation exercises. Country-level implementation evidence was derived primarily from PHEPA assessments conducted pursuant to Article 8 of Regulation (EU) 2022/2371, under which ECDC assesses Member States’ implementation of national prevention, preparedness and response plans on a three-year cycle [1,20].

The interim PHEPA synthesis was retained as a contextual cross-country source. It summarises the first 15 ECDC assessments, 10 of which were also represented by individual country reports in the corpus: Belgium, Estonia, Finland, France, Iceland, Latvia, Lithuania, Luxembourg, the Netherlands and Spain. Cyprus and Liechtenstein were not part of that first group of 15. Because aggregate findings could not be reliably separated by country or matched to individual assessment passages, all 15 synthesis observations (12 with documented gaps) were excluded from every numerical estimate. This conservative whole-source exclusion avoids potential double counting but also omits any distinct information in the synthesis about countries without individual reports in the corpus.

The full extraction matrix contains 190 rows: 168 capability-evidence observations, 21 documented-gap-only observations and 1 context-only threat-framing reference. The interim synthesis (Source ID P01) contributes 15 rows (13 capability-evidence and 2 gap-only), leaving 175 rows from 34 sources for frequency estimates: 155 capability-evidence, 19 gap-only and 1 threat-framing reference. Of these 175 rows, 174 have a readiness-domain assignment; the context-only row is included in the general denominator but not the domain display. The full matrix contains 74 documented-gap rows, of which 12 are from P01; thus, 62/175 (35.4%) eligible rows report an explicit gap. The counting filters and reconciliation formulas are provided in Supplementary Material S4.

### 3.2. Overall Pattern of Operationalisation

At source level, evidence was concentrated in formal policy commitments and established mechanisms, while fewer sources documented exercise testing or real-world use. Of the 34 sources contributing to frequency estimates, 29 contained policy-level evidence (P), 26 documented established mechanisms or resources (M), 19 contained explicit evidence of operational functionality (O), 8 documented completed exercise testing (E), and 8 documented real-world activation or deployment (R). Across the 175 rows in the quantitative subset, the corresponding counts were 103 P, 116 M, 70 O, 17 E and 13 R.

The distribution shows that documentary evidence was substantially more extensive for policy commitments and established mechanisms than for exercise testing or real-world use. Specific instances of use included diagnostic expansion during COVID-19 described in the Cyprus PHEPA [22], EU Health Task Force assignments and field deployments [23], and rescEU medical evacuation and medical/CBRN reserve deployments in response to the war in Ukraine [24]. These documents establish particular activities or deployments; they do not measure the performance of the whole EU/EEA health-security system or establish effectiveness in a future crisis.

**Figure 1:**
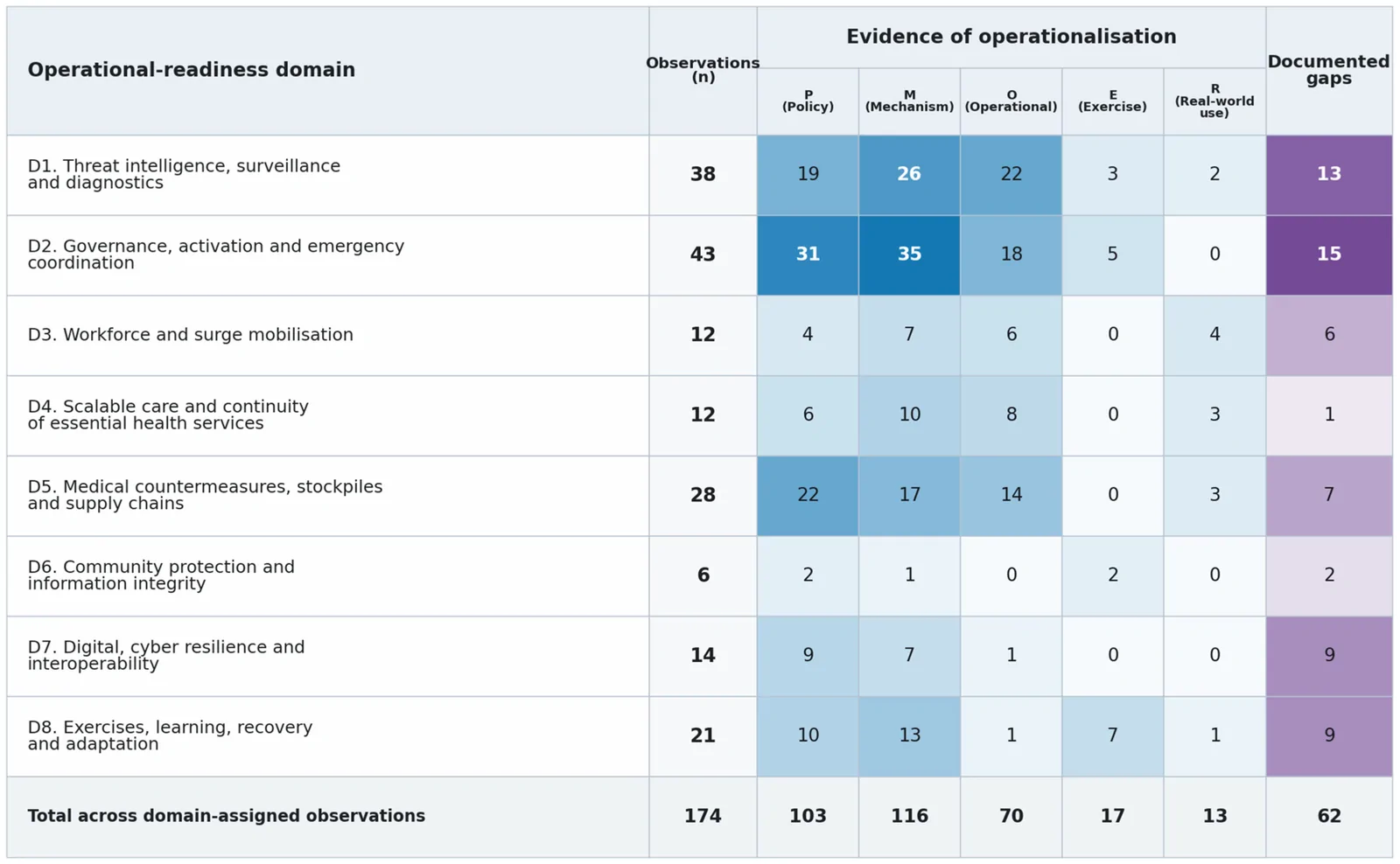
Distribution of evidence-of-operationalisation categories and documented gaps across the eight operational-readiness domains. Note: Blue shading represents the numbers of observations assigned to the P, M, O, E and R evidence categories; purple shading represents the numbers of observations with documented gaps. Darker shades indicate larger counts within each separate colour scale. Grey cells show observation and column totals. Evidence categories are non-mutually exclusive. Counts are based on 174 domain-assigned observations; one additional threat-framing reference was not assigned to an operational-readiness domain.

Documented implementation gaps were identified in 62 of the 175 quantitative coded observations and occurred in 17 of the 34 sources contributing to frequency estimates. Gaps were represented across all eight operational-readiness domains. The distribution of evidence-of-operationalisation categories and documented gaps across the eight readiness domains is summarised in Figure 1.

### 3.3. Threat Intelligence, Surveillance and Diagnostics

Public documentary evidence for threat intelligence, surveillance and diagnostic arrangements was broad at the policy and institutional levels, but evidence of integration and scalability remained uneven. Thirty-eight document–capability observations from 20 primary sources were mapped to this domain. Policy-level evidence was identified in 19 observations, established mechanisms in 26, and explicit operational functionality in 22. Three observations documented completed exercise testing, two documented real-world use, and thirteen contained explicitly documented implementation or capacity gaps.

Surveillance capability increasingly reflected a multi-source and cross-sector model. At the EU level, the emerging architecture combined epidemic intelligence, wastewater and environmental surveillance, genomic and laboratory capacity, and structured health-threat prioritisation. HERA’s 2026 Work Plan continued development of the ATHINA threat intelligence system, support for the Epidemic Intelligence from Open Sources (EIOS) system, wastewater surveillance, and the GLOWACON aviation surveillance programme [25]. ECDC preparedness assessments likewise treated surveillance and laboratory capacity as core preparedness functions [15].

The documentary basis for laboratory and diagnostic activity was specific to particular networks and events. HERA’s 2026 Work Plan described the established DURABLE laboratory consortium and planned further diagnostic, sequencing and reference-laboratory initiatives [25]. The Cyprus PHEPA documented high-volume diagnostic testing during COVID-19 and a tiered laboratory network, while noting that the laboratory preparedness plan still required finalisation and stress testing [22]. National assessments and the first-cycle PHEPA synthesis also identified limitations in formal surge arrangements, specialised laboratory access, trained personnel, specimen referral, data interoperability and sustainability [15,22]. These records document selected functions and past use but do not establish effective or sustained EU/EEA-wide laboratory surge in future crises.

A second recurrent limitation concerned data integration and interoperability. Country assessments documented fragmented surveillance systems, incomplete automation, inconsistent laboratory-to-surveillance data flows, and limited interoperability between information platforms. Cyprus illustrates this pattern. The CIPHIS platform enables participating primary-care clinicians to report electronically, access near-real-time dashboards, and link epidemiological and laboratory data for sentinel surveillance; however, substantial reporting remained manual, automatic extraction from electronic health records was incomplete, and laboratory surveillance remained distributed across multiple information systems [22].

Risk assessment and foresight mechanisms were also represented across the corpus, although their degree of institutionalisation varied. The PHEPA interim analysis documented differences in national preparedness contexts and implementation capacity [15], while EU-level threat prioritisation was more formally structured for medical-countermeasure planning. HERA’s Comprehensive 2025 Health Threat Prioritisation Assessment identified four major categories: respiratory or contact-based viruses with pandemic potential; vector-borne or animal-reservoir viruses with epidemic potential; antimicrobial resistance; and armed-conflict-related and chemical, biological, radiological and nuclear threats [26].

Taken together, the dominant documented limitations in this domain concerned incomplete interoperability, variable formalisation of surge arrangements, and uneven evidence that existing surveillance, laboratory, and risk assessment mechanisms could function as integrated and scalable operational systems.

### 3.4. Governance, Activation and Emergency Coordination

Governance, activation and emergency coordination was the most extensively represented operational-readiness domain, comprising 43 document–capability observations across 22 primary sources. Policy-level evidence was identified in 31 observations, established mechanisms in 35, and explicit operational functionality in 18. Five observations documented completed exercise testing, while no observation met the predefined criteria for real-world-use coding at the governance level. Fifteen observations contained explicitly documented governance or implementation gaps.

At EU level, the corpus documented an increasingly interconnected crisis governance architecture linking health-security mechanisms with wider crisis management, civil-protection and cross-sector coordination arrangements. The 2025 Union prevention, preparedness and response plan for health crises, for example, describes joint capacities and resources that may be mobilised or activated during a Union-level health emergency and explicitly links health response with cross-sectoral and civil-protection mechanisms [27]. At national level, the PHEPA process assessed implementation of emergency management, coordination and preparedness arrangements rather than solely the formal existence of plans, thereby providing an implementation-focused evidence layer for this domain [15,20].

The most recurrent national-level limitation concerned the translation of strategic preparedness into implementable operational arrangements. Documented problems included insufficiently detailed standard operating procedures, unclear activation or escalation thresholds, incomplete alignment between strategic and operational plans, continuing reliance on informal coordination relationships, and emergency-operations or incident management structures that were still under development.

Country assessments also showed that the existence of formal emergency plans did not always ensure alignment across administrative levels. In Belgium, ECDC recommended clearer separation and alignment between the General Preparedness Plan, the Viral Outbreak Plan and federated-entity plans [28].

Cross-border and cross-sector coordination was widely represented in the corpus, although the degree of documented operationalisation varied. Bilateral agreements, EU mechanisms and multisectoral structures provided formal channels for coordinated action, whereas several national assessments identified continued reliance on informal relationships, incomplete standardisation of information exchange, or insufficiently formalised roles and responsibilities. These limitations were particularly evident at interfaces linking public health with civil protection, border authorities, law enforcement and security actors.

Civil–military and deliberate-threat coordination was less extensively documented than general emergency coordination, but completed exercises provided evidence of selected capabilities. Operation Vector, conducted jointly by ECDC and Europol on 11–12 November 2025, involved more than 100 operational and technical professionals and tested multi-country public-health and law-enforcement coordination in a scenario involving the intentional release of a genetically engineered pathogen. The exercise addressed early-warning mechanisms, cross-sectoral information exchange, communication and operational decision making under time pressure [29].

However, such exercise evidence remained selective rather than system-wide. Across the wider corpus, the documented formalisation and exercise testing of civil–military and cross-sector health coordination varied, and several newer EU initiatives had not yet progressed to completed exercise testing by the search closure date. HERA’s 2026 Work Plan, for example, announced two simulation exercises for 2026: one focusing on intentional CBRN threats within a multisectoral approach including civil–military cooperation, and another testing the updated pandemic procurement framework [25]. Because these exercises were prospective in the source document, they were not coded as E evidence.

Taken together, this domain was characterised by a dense formal and institutional architecture, alongside a narrower documentary evidence base for clearly specified activation procedures, repeated exercise testing, and sustained operational coordination across sectors and jurisdictions.

### 3.5. Workforce and Surge Mobilisation

Workforce and surge mobilisation was less extensively represented in the documentary corpus than surveillance or governance, comprising 12 document–capability observations from 10 primary sources. Four observations contained policy-level evidence, seven documented established workforce mechanisms, six demonstrated operational mobilisation functionality, and four provided evidence of real-world activation or deployment. No observation met the predefined threshold for exercise-based evidence (E), while six identified workforce-related limitations.

The strongest operational evidence concerned deployable public-health personnel. The EU Health Task Force (EUHTF) functioned as an established supranational mobilisation mechanism providing preparedness and outbreak-response support on request. According to the EUHTF 2025 Bulletin, 14 requests were accepted during 2025, with assignments in 12 EU/EEA locations and 2 outside the EU/EEA; six experts were deployed [30]. The same source described the ECDC staff and fellowship pools as operational, while the external expert pool remained only partially operational and was being developed incrementally [30]. These findings document established mobilisation capacity while also identifying limitations in workforce scalability.

Country-level evidence showed a similar coexistence of mobilisation capacity and workforce constraints. National assessments documented mechanisms for deploying or redistributing personnel during emergencies, while recurrent limitations included small specialist teams, incomplete workforce mapping, limited reserve capacity, and the difficulty of maintaining routine functions while simultaneously supporting emergency response. Cyprus illustrates this pattern. ECDC reported that the Ministry of Health had demonstrated the ability during COVID-19 to coordinate and mobilise human resources at national and intermediate levels, while also identifying several preparedness functions dependent on very small teams and recommending the creation of trained reserve pools [22].

Workforce continuity represented a distinct challenge from short-term mobilisation. Although deployment or redistribution could be demonstrated in several observations, the documentary evidence for maintaining adequate specialist staffing during prolonged, concurrent or geographically distributed disruptions was more limited. Training and competency development mechanisms were nevertheless present. The EUHTF documented outbreak-response and analytical training activities [30], while HERA’s 2026 programme continued EU-level training, e-learning and staff-exchange activities intended to strengthen preparedness and response competencies [25].

Taken together, the dominant documented workforce limitation concerned the sustainability and scalability of specialist human resources over time, rather than the absence of mobilisation mechanisms themselves.

### 3.6. Scalable Care and Continuity of Essential Health Services

Scalable care and continuity of essential health services showed a high concentration of operational evidence relative to the volume of documentation available. Twelve document–capability observations from eight primary sources were assigned to this domain; six contained policy-level evidence, ten documented established mechanisms, eight described operational functionality, and three documented real-world use. No observation met the predefined threshold for exercise-based evidence (E), while only one contained an explicitly documented capability gap.

The clearest real-world evidence concerned patient redistribution and medical evacuation. Cross-border transfer arrangements were described at both EU and national levels, including the Union Civil Protection Mechanism, and a rescEU medical-evacuation capability that the Commission reports was used in the context of the war in Ukraine [24,27]. In a separate notice, the Commission reported that by April 2026, it had facilitated transfers of more than 5000 patients from Ukraine to hospitals in 22 European countries under the Union Civil Protection Mechanism [31]. This documents completed cross-border transfers, coordination and mobilised transport and receiving hospitals; it does not provide a comparative evaluation of clinical outcomes, effectiveness or resilience under concurrent system disruptions.

The Cyprus PHEPA describes agreements with Greece, Israel and Italy for mutual assistance and patient transfers and states that these agreements were tested in recent evacuations of people from the Middle East and humanitarian operations to Gaza [22]. It does not specify that patient transfers were performed under those agreements during those events; accordingly, the observation was not coded as real-world patient evacuation. The report also records that Cyprus received and provided rescEU assistance, including during wildfires, and that public and private hospital capacity was monitored and managed during COVID-19. ECDC identified incomplete Ministry-level visibility of private-sector bed and workforce capacity as a limitation for whole-system surge situational awareness [22].

Evidence for continuity of essential services was more heterogeneous. At EU level, the Commission’s 2026 critical-infrastructure guidance addressed prevention of disruption to essential services and resilience against natural, man-made, cross-sectoral and cross-border risks, including public-health emergencies and hybrid threats [32]. At national level, sector-specific continuity mechanisms were identifiable; for example, the Cyprus assessment reported operational continuity plans across reference laboratories intended to maintain critical functions and support rapid restoration following disruption [22].

Within the coded D4 observations, however, no capability met the predefined threshold for exercise testing. The documentary evidence was, therefore, stronger for operational patient redistribution, medical evacuation and selected surge or continuity mechanisms than for completed exercises explicitly testing continuity of essential health services under complex cross-sector pressures.

Taken together, this domain revealed a distinction between documented capacity to redistribute patients or mobilise medical transport and the more limited evidence concerning sustained continuity of the broader spectrum of essential health services during prolonged or concurrent disruption.

### 3.7. Medical Countermeasures, Stockpiles and Supply Chains

Public documentation provided substantial evidence of established and operational medical-countermeasure, stockpiling and supply-chain arrangements, although important limitations remained in demand visibility and scalable manufacturing. Twenty-eight document–capability observations from fourteen primary sources were mapped to this domain. Policy-level evidence was identified in 22 observations, established mechanisms in 17, and explicit operational functionality in 14. No observation met the predefined threshold for exercise-based evidence (E), while three documented real-world use and seven contained explicitly documented implementation or capacity gaps.

The distribution of evidence varied across the four indicators. Strategic reserves were represented by four observations from four sources, while inventory and needs visibility accounted for six observations from six sources. Procurement and manufacturing surge was the most frequently represented component, with 10 observations from seven sources, followed by logistics and deployment with 8 observations from seven sources. One documented gap concerned strategic reserves, two inventory and needs visibility, and four procurement and manufacturing surge; no explicit gap was coded for logistics and deployment in the available evidence.

At EU level, the medical-countermeasure architecture increasingly addressed the full preparedness-to-deployment cycle. The 2025 Medical Countermeasures Strategy linked surveillance and intelligence, research and development, scalable manufacturing, procurement, stockpiling and rapid deployment within an end-to-end approach. It also set out flexible and scalable manufacturing arrangements, including RAMP-UP, together with reserved production capacity, improved stockpiling practices and crisis procurement arrangements [4].

Strategic stockpiling showed explicit operational and real-world evidence. The rescEU reserve included strategic medical and CBRN stockpiles across participating Member States; on the Commission page accessed in September 2026, the Commission reported that 17 of 22 stockpiles under development were fully operational [24]. The 2025 EU Stockpiling Strategy further broadened the approach by addressing the full stockpiling cycle, including anticipation, supply-chain monitoring, identification of gaps and duplications, stock management, transport and deployment [33].

Real-world evidence also documented the use of selected EU-level procurement and deployment mechanisms. During the 2022 mpox outbreak, the Commission secured more than 109,000 vaccine doses in June 2022, which were distributed to Member States and associated countries within a month. In 2023–2024, subsequent joint procurement secured access to up to two million doses, allowing participating countries to build stocks and plan vaccination strategies in advance [27]. These observations were coded as real-world evidence only for the specific procurement and deployment capabilities involved and were not interpreted as evidence of readiness across the medical-countermeasure system as a whole.

National assessments documented a different recurrent pattern. Physical strategic stockpiles were widely present, while mechanisms for determining stock composition and volumes, monitoring supply and demand, linking risk scenarios to demand estimates, and planning manufacturing scale-up were less consistently developed [15]. In the first 15 PHEPA assessments, for example, supply-and-demand monitoring was frequently fragmented or crisis-activated, scenario-based demand forecasting was limited, and systematic mapping of manufacturing capacity was incomplete.

Belgium’s assessment recommended continued development of tools for monitoring medical-countermeasure supply and estimating demand, together with clearer federal–regional reporting responsibilities [28].

Taken together, the dominant documented limitation was not the absence of medical reserves but the integration of those reserves into an end-to-end capability able to anticipate requirements, maintain visibility of inventories and supply constraints, scale production, replenish stocks and deploy countermeasures rapidly under changing or simultaneous crisis conditions. EU-level policy increasingly addressed these functions, while national implementation evidence remained heterogeneous.

### 3.8. Community Protection and Information Integrity

Community protection and information integrity were the least extensively documented of the eight operational-readiness domains. Six document–capability observations from six primary sources were mapped to this domain. Two contained policy-level evidence and one documented an established mechanism; no observation met the predefined criteria for operational functionality (O) or real-world use (R), while two documented completed exercise testing (E). Two observations contained explicitly documented gaps.

The evidence was concentrated in risk communication and information integrity, with three observations mapped to each. No observation was independently coded to the community-engagement or population-protection indicators.

At EU policy level, information integrity was increasingly incorporated into preparedness planning. The Preparedness Union Strategy included strategic communication, public awareness measures and tools for countering information manipulation within its whole-of-society preparedness approach [3]. HERA’s 2026 Work Plan similarly included measures intended to strengthen knowledge and skills for addressing health-related misinformation and disinformation, including professional networks and communication activities [25].

National implementation evidence, however, indicated that risk-communication, community-engagement and infodemic-management arrangements were not uniformly institutionalised. Spain’s PHEPA assessment identified the need for a more formalised and structured approach to risk communication, community engagement and infodemic management (RCCE-IM), including strategies, standard operating procedures, scalable emergency mechanisms and dedicated trained staff [34].

No separate document–capability observation was coded to population-protection measures. The first-cycle PHEPA synthesis nevertheless documented that, in several assessed countries, decision-making, implementation and evaluation of public-health and social measures remained ad hoc and did not consistently involve all relevant stakeholders [15]. This illustrates the limited public operational evidence available for this aspect of community protection.

Taken together, this domain had a markedly thinner public documentary evidence base linking risk communication, community engagement, information integrity and population-protection arrangements to operational, exercised or real-world performance. This documentary asymmetry limited the extent to which societal and communication capabilities could be assessed using the same evidence thresholds applied to more observable mechanisms such as stockpiles, laboratories, deployments and crisis-coordination structures.

### 3.9. Digital, Cyber Resilience and Interoperability

Public documentary evidence for digital, cyber and interoperability arrangements comprised 14 document–capability observations from 13 primary sources. Policy-level evidence was identified in nine observations, established mechanisms in seven, and explicit operational functionality in one observation concerning cross-sector information exchange. No observation met the predefined criteria for completed exercise testing (E) or real-world use (R) within this domain. Nine observations contained explicitly documented implementation or capability gaps.

The evidence was strongly concentrated in data interoperability. Ten observations were mapped to this indicator, nine of which contained an explicitly documented gap. Across national assessments, digital surveillance and laboratory-information systems were present, but fragmentation between platforms, incomplete automation, inconsistent data-sharing arrangements and limited interoperability remained recurrent constraints.

National assessments illustrated different manifestations of this problem. Luxembourg’s PHEPA identified limited interoperability between databases and digital systems as a potential constraint during major health crises [35]. Belgium was continuing development of the be.Prepared platform for automated integration of laboratory, epidemiological and genomic data, with the ECDC recommending further development and finalisation [28].

The emerging Epi+ architecture provided a further example of digital capability still under development. The ECDC describes Epi+ as a multi-hazard, open-source, event-based surveillance platform designed to support national signal detection, risk assessment, response coordination and documentation before and alongside EU-level information exchange [36]. The ECDC also reported that most Member States did not yet have a dedicated national digital tool for these functions. Testing of the common ECDC platform was scheduled to begin in September 2026, while the first national systems were planned to go live in June 2027, beyond the analytical period of the present study [36]. Accordingly, the D7 observation was not coded as demonstrated operational functionality.

Cybersecurity showed a different evidence pattern. The 2025 European Action Plan on the Cybersecurity of Hospitals and Healthcare Providers established a dedicated sectoral framework centred on prevention, threat detection, preparedness, response and recovery and proposed tailored guidance, tools, services and training, including a pan-European cybersecurity support function [37]. A separately dated 2026 Commission roadmap listed additional subscription, assessment and response services as planned measures, coded as policy commitments rather than established mechanisms [38]. ENISA subsequently signed a three-year contribution agreement with the Commission, published updated healthcare cybersecurity procurement guidance and began developing a service catalogue for the proposed European Cybersecurity Support Centre [39]. The agreement and published guidance support mechanism-level implementation, but this source does not document activation or deployment of a health-sector cyber response service. No cyber response observation met the O, E or R threshold in this corpus.

Artificial intelligence appeared primarily as a cross-cutting modifier rather than as a separately demonstrated health-readiness capability. The Commission’s 2026 Action Plan on Cybersecurity and Artificial Intelligence explicitly described AI as dual-use: advanced AI can support vulnerability detection, cyberattack prevention and critical-infrastructure protection, while malicious actors can use it to automate attacks, identify weaknesses and increase the speed and scale of cyber operations [40]. Within the present corpus, this constituted primarily policy-level evidence concerning an enabling technology and an evolving threat amplifier rather than completed testing of AI-enabled health-crisis capabilities.

No independent observation was coded to digital or communication continuity.

Taken together, the digital domain showed a pronounced documentary asymmetry. EU/EEA health-security architecture increasingly incorporated digital surveillance, interoperability and cyber-resilience mechanisms, but evidence of operational functionality remained substantially thinner than evidence of policy commitments and established mechanisms. The predominant documented constraint concerned the integration of heterogeneous systems, automation of information flows and maintenance of interoperable situational awareness across organisations and sectors during crises.

### 3.10. Exercises, Learning, Recovery and Adaptation

Exercises, learning, recovery and adaptation constituted one of the domains in which preparedness arrangements could be assessed against directly observable exercise testing and learning activity. Twenty-one document–capability observations from 15 primary sources were mapped to this domain. Policy-level evidence was identified in 10 observations, established mechanisms in 13, explicit operational functionality in 1, completed exercise evidence in 7, and real-world evidence in 1. Nine observations contained explicitly documented gaps.

Completed exercise reports supplied the clearest direct testing evidence in this domain. The PHEPA interim synthesis indicated that approximately half of the first 15 assessed countries regularly conducted simulation exercises, although the frequency, scope and institutionalisation of national exercise programmes varied [15]. Country-level assessments documented regular or repeated exercises in several settings, while EU Health Task Force support contributed additional simulation activity during 2026.

Completed exercises also provided direct evidence of selected capabilities beyond cross-sector coordination. In Belgium, a March 2026 Ebola-based simulation explicitly tested information exchange, surveillance and early detection of imported cases, and risk-communication approaches. The exercise was coded separately as completed exercise evidence for surveillance and early detection (D1.1), laboratory diagnosis and diagnostic preparedness (D1.2), cross-sector coordination (D2.3), risk communication (D6.1), and the exercise programme itself (D8.1) [41,42].

Operation Vector in November 2025 was a completed exercise of a deliberate biological-release scenario. It explicitly tested early-warning mechanisms, cross-sectoral information exchange, public risk communication, coordination and decision making. Completed exercise evidence was recorded separately for early warning (D1.1), cross-sector coordination (D2.3), risk communication (D6.1) and the exercise programme (D8.1). The scenario involved one initiating biological hazard; working across several agencies did not make it a hybrid campaign or compound crisis [29].

A marked difference emerged between conducting exercises and institutionalising learning from them. After-action learning accounted for three observations, two of which contained documented gaps. The PHEPA interim evidence identified inconsistent use of structured after-action reviews following real events [15], while national assessments showed that lessons from exercises or emergencies were not always systematically captured, prioritised or translated into formal corrective-action processes. Lithuania, for example, reported frequent participation in multi-stakeholder exercises but was advised to develop a more strategic cross-sector exercise programme and a systematic process for integrating lessons into national emergency planning [43].

ECDC’s 2026 guidance reinforces this distinction by treating the action review as part of a broader cycle, extending from preparation and review through follow-up. The guidance specifies that identified findings and proposed corrective actions should be consolidated into a final report and translated into an action plan for implementation [44].

Real-world learning was nevertheless identifiable in selected observations. In Cyprus, internal reflection following COVID-19 led to changes in surveillance arrangements despite the absence of a formal AAR process [22]. Lithuania similarly documented technical and structural changes resulting from previous events and exercises, including virtual meetings, digitalised procedures and tiered activation of emergency operation centres [43]. At EU level, the EU Health Task Force supported countries not only through simulation exercises but also in translating preparedness assessments into plans and follow-up actions, including support for Luxembourg’s post-PHEPA action plan [30].

More recent ECDC activity provided direct examples of structured learning following real events. In March 2026, ECDC and the EU Health Task Force completed an after-action review with Latvian authorities following the 2025 leptospirosis outbreak [45]. In April 2026, the EUHTF also supported an after-action review in Slovakia following the 2025 multi-country hepatitis A outbreak, involving participants from Slovakia, Austria and Czechia [46]. These examples demonstrate operational use of formal review mechanisms, although such practices were not uniformly documented across the national assessments analysed.

### 3.11. Threat Contexts in Public Documentation

Threat-context coding described the framing of all 190 extracted observations, including the contextual P01 rows; it did not assess documented operational readiness for particular crisis configurations. Intent was coded natural/accidental in 40 observations, deliberate in 15 and unclear in 135. Interaction was coded single hazard in 49, multi-hazard in 19 and not specified/not applicable in 122. No row met the strict threshold for a hybrid campaign, compound crisis or cascading disruption. A consistency check found a permitted intent and interaction value in every matrix row; this checks application of the stated categories but is not independent validation.

The matrix records seven boundary-case threat-framing notes (OBS057, OBS065, OBS100, OBS104 and OBS109–111). A strategy and critical-infrastructure guidance name hybrid threats without describing the coordinated malign activity and combined instruments required for a hybrid-campaign code (OBS057, OBS109) [32,33]. Planned measures addressing misinformation and AI-enabled cyberattack risk likewise do not establish such a campaign (OBS100, OBS104). Operation Vector tested a deliberate biological incident but did not describe interacting hazards or a combined-instrument campaign (OBS065) [29]. References to essential-service protection and recovery do not describe an initiating event causing secondary failures that affect health-system functioning (OBS110–111) [32]. Multi-hazard codes instead identify explicit all-hazards scope or multiple distinct hazard classes; they do not imply a compound crisis. The source locators and decisions for these cases are shown in Supplementary Material S4.

Accordingly, the finding concerns the limited public visibility and specification of complex threat configurations in the included documents. It does not support an inference about the presence or absence of EU/EEA operational readiness for hybrid, compound or cascading crises.

### 3.12. Cross-Cutting Enablers, Dependencies and Scalability

Across the eight readiness domains, operational capability depended on cross-cutting conditions that were not treated as stand-alone health capabilities within the analytical framework. Legal authority and mandate, flexible emergency financing and resource mobilisation, and evidence-to-policy or decision support were coded as cross-cutting enablers. Workforce, logistics, transport, manufacturing, supply chains, laboratory capacity, digital infrastructure, civil protection and inter-organisational coordination recurred as operational dependencies across multiple domains.

Legal authority and mandate were most visible in relation to activation, coordination and the formalisation of responsibilities. Several national assessments described functional arrangements that depended partly on established practice, voluntary arrangements or informal networks rather than fully specified statutory or procedural mechanisms. Cyprus, for example, continued to operate under an older infectious-disease legal framework while legislative reform was underway, and the ECDC identified incomplete formalisation of cross-ministerial communication, escalation procedures and continuous public-health coordination functions [22]. The evidence, therefore, distinguished between the practical existence of coordination mechanisms and the legal or procedural formalisation required to make responsibilities, authority and activation arrangements explicit during emergencies.

Flexible emergency financing represented a second enabling condition. Where documented, financing arrangements could support rapid mobilisation. In Cyprus, state-budget reserves and supplementary-budget mechanisms could be activated for urgent needs, and the ECDC reported that resources had been released within 24–48 h during COVID-19 and the 2025 wildfires [22]. At the same time, Cyprus had no dedicated budget line for IHR implementation or preparedness activities, no real-time mechanism for monitoring IHR expenditure, and no systematic testing of contingency financing for public-health emergencies [22]. This illustrates the distinction between the ability to release funds during an emergency and the availability of predictable financing for maintaining workforce, laboratory, digital and stockpiling capabilities between crises.

Evidence-to-decision support was represented across surveillance, risk assessment, medical-countermeasure prioritisation and situational-awareness mechanisms. Risk assessments, epidemic intelligence, laboratory information, supply monitoring and crisis-information systems linked preparedness information with operational decision processes. However, national assessments repeatedly documented fragmented datasets, incomplete automation and limited visibility across organisational or public–private boundaries. The existence of information-generating mechanisms, therefore, did not necessarily imply fully integrated or timely decision support across the system.

Dependency coding further showed that operational capabilities were rarely self-contained. Laboratory surge depended on specialist personnel, reagents, digital systems and specimen transport; medical-countermeasure capability depended on manufacturing, procurement, logistics and cross-border movement; clinical surge depended on workforce availability, transport and visibility of receiving capacity; and digital capability depended on communications infrastructure, cybersecurity and institutional interoperability. Contemporary EU critical-infrastructure policy reflects similar interdependencies: the Commission’s 2026 guidance addresses cross-sectoral and cross-border risks, public-health emergencies, hybrid threats, disruption of essential services, and mitigation and recovery from disruptive incidents [32].

Cross-border scalability provided a further distinction between the existence of a capability and evidence that it could be used beyond its immediate institutional or national setting. Explicit cross-border scalability was documented in 37 of the 175 quantitative observations across 17 of the 34 sources. In the remaining 138 observations, cross-border scalability was not publicly documented.

Governance coding showed a similarly layered architecture. Of the 175 quantitative observations, 91 were coded at national governance level, 43 as shared arrangements and 41 at EU level. National systems, therefore, remained central to operational delivery, while selected functions, including medical-countermeasure procurement and stockpiling, deployable workforce mechanisms, medical evacuation and crisis coordination, were supported through shared or EU-level arrangements [24,27,30].

The cross-cutting analysis consequently indicated that documented operational readiness depended not only on the existence of individual health capabilities but also on whether the dependencies required for their activation and sustained use were available. The operational value of a capability could, therefore, vary according to workforce availability, transport access, digital and communications continuity, laboratory capacity, supply-chain resilience, legal authority and the ability to mobilise resources across organisational or national boundaries.

Taken together, scalability and interdependence emerged as central analytical properties of operational readiness rather than secondary characteristics of preparedness. Formally established capabilities whose operation depended on poorly documented, untested or fragile external systems could not be assumed to retain equivalent functionality during prolonged, cross-sectoral or cascading disruption.

### 3.13. Preparedness–Readiness Gaps

Documented preparedness–readiness gaps were identified in 62 of the 175 analytical observations contributing to quantitative estimates, originating from 17 of the 34 sources. Gaps were present across all eight readiness domains but were unevenly distributed: governance, activation and emergency coordination accounted for 15 gap observations; threat intelligence, surveillance and diagnostics for 13; digital, cyber resilience and interoperability and exercises, learning, recovery and adaptation for 9 each; medical countermeasures, stockpiles and supply chains for 7; workforce and surge mobilisation for 6; community protection and information integrity for 2; and scalable care and continuity of essential health services for 1.

Six recurrent gap mechanisms, summarised in Table 3, described documented problems of formalisation, interoperability, scalability, exercise or event testing, learning and corrective-action closure, and cross-sector or cross-border integration.

Formalisation and activation gaps were reported where strategic plans and coordination structures lacked detailed procedures, activation thresholds, clear responsibilities or completed emergency-operations arrangements. The PHEPA interim synthesis noted implementation challenges alongside established preparedness strengths while emphasising variation in national context and implementation [15].

Interoperability and visibility gaps concerned integration of surveillance, laboratory, hospital-capacity and medical-countermeasure information. National assessments reported database-interoperability limitations and ongoing development of integrated surveillance platforms; comparable issues affected stock monitoring, demand projection and supply-chain coordination [28,33,35,36].

Scalability and sustainability gaps concerned the ability to expand and maintain capability under prolonged or simultaneous pressure. The corpus documented EU-level deployment capacity but a still-expanding external expert pool [30] and identified manufacturing, procurement, supply-chain resilience, logistics, workforce, consumables and receiving capacity as conditions for sustained countermeasure, laboratory and clinical surge [4,25,33].

Completed exercises documented selected outbreak management, multisector and deliberate-biological capabilities, including Operation Vector and exercises in Belgium and Malta [29,41,42]. Publicly documented exercise, after-action or real-world evidence was less available for several arrangements assigned to the exercise and event evidence mechanism, while hybrid, compound and cascading terms were usually threat framing rather than completed scenario testing [37,39].

Learning and corrective-action gaps were reported where lessons from exercises or events were not systematically incorporated into preparedness plans. The PHEPA synthesis and country evidence highlighted inconsistent after-action review and follow-up [15,43], although ECDC guidance and documented reviews in Latvia and Slovakia demonstrate that structured learning and corrective action are feasible [44,45,46].

Cross-sector and cross-border integration gaps involved incomplete formal interfaces, reliance on informal information exchange and limited public evidence of external scalability. The corpus nevertheless contained examples of deliberate-threat coordination and medical evacuation [24,27,29,31,32].

Because PHEPA assessments constituted the principal source of national-level implementation evidence, a sensitivity analysis was conducted to examine whether the identified preparedness–readiness gap patterns were disproportionately driven by PHEPA-derived observations. The 175 analytical observations were, therefore, stratified into PHEPA-derived and non-PHEPA evidence streams and compared descriptively with respect to the frequency and domain distribution of documented gaps and the six recurrent gap mechanisms identified above. No statistical testing was performed. The results are presented in Table 4.

PHEPA sources contained 55 documented gaps among 100 observations (55.0%), compared with 7 among 75 non-PHEPA observations (9.3%). The difference may reflect source function, reporting depth and composition and is not comparative evidence of documented operational readiness at the national versus EU level. PHEPA gaps spanned eight domains, whereas non-PHEPA gaps were found in four; three of six mechanisms were present in both streams, but only seven non-PHEPA gaps were available. Of 62 gap observations, 52 did not contain evidence of operational functionality, completed exercise testing or real-world use; the remaining 10 did, showing that documented limitations can coexist with other forms of operational evidence.

Excluding Cyprus left 148 observations and 44 documented gaps (29.7%); excluding Cyprus and Liechtenstein left 136 observations and 35 gaps (25.7%). In both analyses, interoperability and visibility and scalability and sustainability remained the most frequent mechanisms; D4 had no documented gap after Cyprus was omitted (Table 5).

### 3.14. Empirical Distribution of Recurrent Preparedness–Readiness Gaps

**Figure 2:**
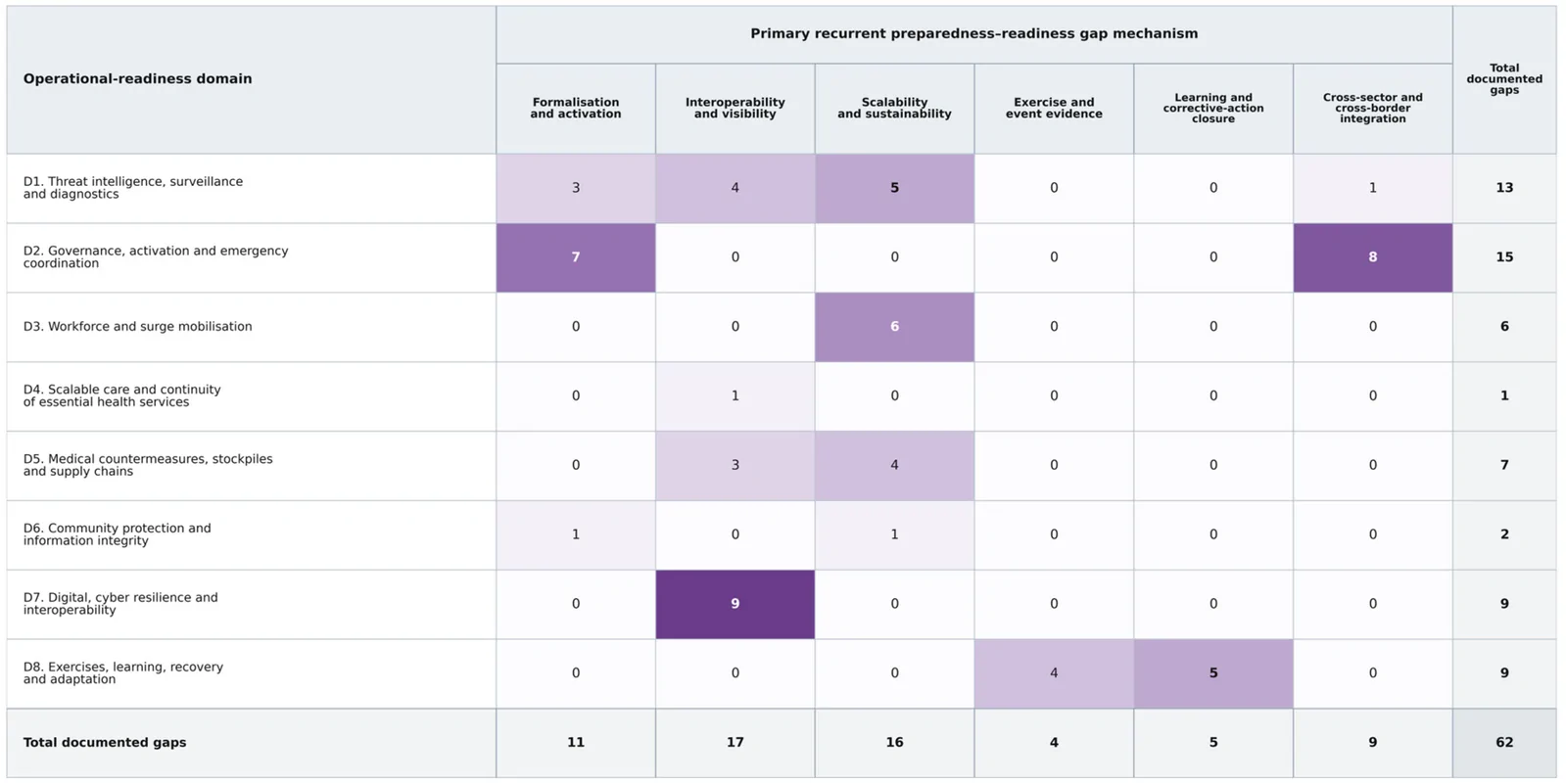
Distribution of primary recurrent preparedness–readiness gap mechanisms across the eight operational-readiness domains. Legend: Cells show the number of observations with an explicitly documented gap assigned to the primary recurrent gap mechanism in Supplementary Material S4. Darker purple shading indicates larger counts; cells marked 0 have no observation assigned to that mechanism in that domain. Grey cells show row and column totals and are not part of the colour scale. Each documented gap was assigned to one primary mechanism. The figure depicts patterns in the documentary corpus and does not measure comparative operational readiness or the prevalence of system deficits.

The predefined framework organised extraction and interpretation; it was not derived as a new result. Figure 2 maps the primary recurrent gap mechanism assigned to each documented-gap observation across the eight operational-readiness domains.

Figure 2 is descriptive and non-causal. It indicates where public sources reported gap mechanisms; it neither describes a mandatory sequence of evidence types nor establishes absent or ineffective capability.

Interpretation of the map considers cross-cutting enablers (legal authority, financing and evidence-to-decision arrangements), dependencies (workforce, digital systems, energy, transport, laboratories and supply chains), cross-border scalability, and learning from exercises and events. These are transversal conditions rather than additional readiness domains [3,4,19,32,33,44].

## 4. Discussion

This analysis maps patterns in public documentary evidence rather than directly measuring system-wide operational readiness. Across the eight domains, formal plans, mechanisms and resources were more frequently documented than activation, completed exercises and real-world use. Differences in source purpose matter: PHEPA reports are implementation-oriented and record strengths and shortcomings, while many non-PHEPA documents are EU strategies or work programmes. The results, therefore, show where public operational evidence is limited or assessments report challenges, not a comparative prevalence of documented operational-readiness gaps at national versus EU level.

Unlike the governance analysis in [6], this study codes source-located document–capability observations across eight domains. Its nonexclusive P/M/O/E/R attributes distinguish policy commitments, mechanisms, reported operation, completed testing and real-world use; a separate field distinguishes reported gaps from absent disclosure. National PHEPA assessments add implementation findings, while source-sensitivity analysis reveals their influence on the mapped gaps. The shared codebook and matrix make these judgements inspectable for later documentary assessments, subject to search and single-coder limitations; they cannot establish whether the reforms improved emergency performance.

This distinction aligns with empirical COVID-19 findings, which show that the association between pre-pandemic preparedness indices and outcomes depends on the outcome and adjustment strategy [13,47]. P/M/O/E/R does not measure effectiveness or predict outcomes; it distinguishes the forms of public evidence available between formal commitments and potential performance.

Across domains, the strongest activity records remained specific to particular functions and settings. The documents describe past diagnostic expansion and selected surveillance tools, alongside continuing laboratory-planning and interoperability gaps [15,22,25,28,35,36]. In cyber resilience, the cited sources document a sectoral policy framework, an agreement and published procurement guidance, rather than an activated health-sector cyber response service [37,39]. Commission notices document cross-border medical evacuation from Ukraine at scale but give no comparative performance measure or evidence of continuity during a prolonged, system-wide shock [24,31].

Within this documentary analysis, medical-countermeasure readiness is better understood as a supply system than a stockpile count: reserves require visibility of demand and stocks, manufacturing, procurement, logistics, cross-border movement and replenishment [4,25,33]. The same dependency logic applies across the corpus. Workforce, transport, finance, laboratories, digital systems and infrastructure condition whether nominally established capabilities retain function under pressure [11,12]. This was why this study required explicit evidence of interaction or causal propagation before coding compound or cascading disruption.

At the observation level, most descriptions did not specify hazard interaction, while a smaller set explicitly framed all-hazards, multi-hazard or one-hazard contexts. No extracted observation demonstrated the strict hybrid, compound or cascading configuration. These results concern public documentary framing and disclosure, not performance under those conditions. Operation Vector documented an intentional biological exercise rather than hybrid-health readiness, and AI was treated as a modifier of enabled or disrupted functions rather than a stand-alone readiness capability [29,40].

The E code records that a specified capability was tested in a completed exercise; exercise completion does not establish adequate performance or show that shortcomings were resolved. Evidence for after-action review, corrective-action monitoring and closure was less frequent; structured learning and implementation of remedial actions are, therefore, central to adaptive capability [15,19,43,44,45,46,48,49,50].

PHEPA adds an implementation-oriented perspective below the EU institutional level, but the sensitivity analysis shows source dependence rather than independent confirmation of gap prevalence. PHEPA and non-PHEPA sources differ in function, depth and governance level, and the seven non-PHEPA gaps are too few to establish an independent distribution.

Supplementary Material S3 is a methodological contribution alongside the substantive mapping. Its domain and indicator definitions, source hierarchy, and rules for P/M/O/E/R evidence, dependencies, documented gaps, threat contexts and ambiguous cases make the coding decisions inspectable. The codebook could be reused in future PHEPA cycles or other public-document assessments to track changes in the type of evidence available for a capability. Prospective searches and independent coding would help test its reliability in those settings; repeated use would still describe documentary evidence rather than yield a direct readiness score.

Future implementation-focused assessments should test interacting capabilities under realistic pressure, including interoperability, supply and workforce scalability, cross-border coordination and dependencies affecting continuity of care [3,25,44]. They should trace exercise or event findings to assigned, resourced and monitored corrective actions. Applying the codebook in repeated reviews would track shifts in public evidence without inferring operational readiness from a plan, institution or stockpile or ranking heterogeneous systems. Future purposively sampled EU/national interviews and exercise reviews could assess activation, resources and cross-sector coordination in practice.

## 5. Limitations

First, the analysis relied primarily on public institutional documents. Some operational capabilities may be recorded only internally or withheld for security, operational or political reasons, particularly civil–military coordination, cyber response, CBRN preparedness, critical-infrastructure protection and deliberate-threat scenarios. Absence of public evidence was, therefore, distinguished from an explicitly documented capability gap. No key-informant interviews or direct observations were undertaken, so the analysis could not independently determine how actors activate, resource or coordinate these arrangements in practice.

Second, sources differed in purpose, detail and reporting depth. Legislation, strategies, work programmes, country assessments, exercise records and deployment reports are not equivalent indicators of underlying readiness. The P/M/O/E/R framework distinguishes their evidence types but cannot remove differences in disclosure: a capability may function without a public exercise or deployment report, whereas visible mechanisms may dominate because they generate fuller documentation.

Third, PHEPA reports are implementation-oriented external assessments that document strengths and shortcomings, while non-PHEPA sources consist mainly of EU strategies, work programmes, operational reports and institutional communications. Source function is closely linked to governance level. The higher frequency of documented gaps in PHEPA reports cannot establish that gaps are more prevalent nationally than at EU level. The seven non-PHEPA gaps also cannot independently confirm the distribution of named mechanisms.

Fourth, several initiatives—including the Preparedness Union Strategy, Medical Countermeasures Strategy, new cyber-health measures, Epi+, HERA programmes and national post-PHEPA actions—were still being implemented when the search closed on 17 September 2026. Mechanisms recorded as policy commitments or under development may later have been activated, exercised or deployed. The findings are, therefore, time-bound.

Fifth, documentary evidence is not a direct measure of performance. An established mechanism, mobilisation procedure or completed exercise cannot establish how effectively a capability would function under future crisis conditions. Conversely, poor performance in one event need not imply a persistent absence of readiness. In particular, E records completed testing, not adequate performance. The framework assesses evidence of operationalisation rather than response quality or outcomes.

Sixth, evidence categories are non-ordinal and were not combined into a readiness score. P/M/O/E/R frequencies reflect the composition of the corpus, not absolute capability strength or comparative readiness across domains or countries. Documented-gap counts also depend on assessment depth and transparency: extensively assessed capabilities may yield more reported weaknesses than those for which less public information exists.

Seventh, most observations did not specify a hazard interaction, and none met the revised threshold for a hybrid campaign, compound crisis or cascading disruption, although deliberately caused biological and CBRN threats appeared. Limited configuration-specific detail may reflect disclosure practices, source purpose or a lack of explicit descriptions. These counts describe public framing and cannot establish whether EU/EEA systems possess the relevant capabilities.

Eighth, community engagement, information integrity and some digital or communication continuity functions yielded less operational evidence than laboratories, stockpiles or deployments. This may reflect observability bias: material resources and activities are easier to document than relational, behavioural or societal capabilities. Future assessments may need tailored indicators and sources.

The original search record lacks individual query dates, candidate and exclusion counts, and record-level decisions; saturation was not assessed. The retained-source inventory supports scrutiny of selected sources but cannot fully reproduce screening or quantify selection bias. The extraction and reported frequencies can be recalculated from the retained matrix, but this does not retrospectively reconstruct the original search and screening decisions.

Original screening, extraction and primary coding were undertaken by one researcher. No independent duplicate screening or coding was completed, and no agreement statistic is available. Domains, P/M/O/E/R evidence attributes, gap mechanisms and threat classifications all involve judgement. The later single-researcher audit changed many intent and interaction assignments, illustrating this sensitivity; its rule and row-level decisions are disclosed in Supplementary Materials S3 and S4, but transparency is not inter-rater reliability. The findings should, therefore, be read as a documentary mapping, not an independently validated estimate of category frequencies or operational capability. This study identifies public evidence and explicitly reported gaps; it does not measure latent capacity or effectiveness.

## 6. Conclusions

This study maps public documentary evidence linking the EU/EEA preparedness architecture to operationalisation. Policy commitments and established mechanisms were more often documented than activation, completed exercise testing, sustained scalability or real-world use.

The mapped gaps are documentary findings rather than a system-wide diagnosis. They were concentrated in PHEPA reports, whose purpose and depth differ from EU-level sources. They identify priorities for implementation-focused assessment, including formalisation, interoperability, scalability, corrective-action closure and cross-border integration, but cannot be read as comparative deficits in underlying operational readiness.

The majority of extracted observations did not state a hazard interaction, and none met the strict hybrid, compound or cascading thresholds. This finding concerns the specificity of public documentary framing and cannot establish the presence or absence of capability for complex crises.

The framework offers a transparent, non-ordinal way to distinguish public evidence types and to track whether documented arrangements are tested, used and followed by corrective action over time.

## Figures and Tables

**Table 1:** Operational Readiness Domains and Analytical Scope.

Domain	Operational Scope
D1. Threat intelligence, surveillance and diagnostics	Integrated surveillance; laboratory and diagnostic surge; risk assessment and foresight; early warning and situational awareness
D2. Governance, activation and emergency coordination	Activation procedures; emergency coordination structures; cross-sector and cross-border coordination; civil–military coordination
D3. Workforce and surge mobilisation	Reserve/deployable workforce; rapid mobilisation and redistribution; emergency competencies and training; workforce continuity
D4. Scalable care and continuity of essential health services	Clinical surge capacity; patient redistribution and evacuation; continuity of essential services; infrastructure redundancy
D5. Medical countermeasures, stockpiles and supply chains	Strategic reserves; inventory and needs visibility; scalable procurement/manufacturing; logistics and rapid deployment
D6. Community protection and information integrity	Risk communication; community engagement; population-protection measures; misinformation/disinformation and information manipulation
D7. Digital, cyber resilience and interoperability	Data interoperability; cyber prevention and detection; cyber incident response and recovery; resilient digital and communication infrastructure
D8. Exercises, learning, recovery and adaptation	Exercise programmes; after-action learning; corrective action and adaptation; documentary framing of hybrid, compound and cascading threats where explicitly addressed

**Table 2:** Composition of the Primary Documentary Corpus and Analytical Sample.

Characteristic	*n*
Primary analytical sources (full corpus)	35
Sources contributing to quantitative estimates	34
EU-level policy/operational sources	22
PHEPA country-level reports	12
PHEPA cross-country interim synthesis (contextual only)	1
Sources dated 2024	1
Sources dated 2025	17
Sources dated 2026	17
Extracted observations (full matrix)	190
Observations included in quantitative frequency estimates	175
Supporting/contextual or methodological sources	3
Foundational/benchmark sources	7
Total retained documentary sources	45

Note: The cross-country PHEPA interim synthesis and its 15 observations, including 12 documented gaps, were retained in the full matrix for transparency but excluded from quantitative estimates because the source overlaps with individual country PHEPA reports. Source-year counts refer to the documentary date recorded in the corpus; for dynamic web resources, the recorded date corresponds to the documented search date or access date where applicable. Source-level categories are descriptive and do not constitute measures of readiness. The complete documentary inventory is provided in Supplementary Material S1 and the full observation-level dataset in Supplementary Material S4.

**Table 3:** Recurrent preparedness–readiness gaps identified across the EU/EEA evidence base.

Recurrent Gap Mechanism	Documented Pattern	Primary Domains Affected	Readiness Implication
Formalisation and activation	Strategic plans not consistently translated into detailed operational procedures; unclear activation triggers; incomplete formal emergency-coordination arrangements	D1, D2, D6	Capability may be conceptually or institutionally established, but activation can depend on ad hoc interpretation or informal arrangements
Interoperability and visibility	Fragmented surveillance and laboratory data; incomplete automation; limited visibility of stock, workforce or service capacity	D1, D4, D5, D7	Decision-makers may lack integrated and timely information required for coordinated action
Scalability and sustainability	Limited reserve workforce; laboratory surge constraints; supply dependencies; insufficient arrangements to sustain capacity as demand increases or persists	D1, D3, D5, D6	Capability may function under baseline or short-duration demand but become constrained as demand increases or persists
Exercise and event evidence	Explicitly reported gaps in completed exercise testing, after-action review or real-event evidence for a specified arrangement	D8	The specified arrangement has not been adequately tested or reviewed in the reported context; no conclusion about successful performance follows from an exercise alone
Learning and corrective-action closure	Inconsistent AAR/IAR use; lessons not systematically tracked; recommendations not linked to monitored implementation	D8	Exercises and events may identify weaknesses without reliably producing capability change
Cross-sector and cross-border integration	Incomplete cross-sector or cross-border interfaces; reliance on informal information exchange; limited evidence of externally scalable coordination	D1, D2	Capability may not retain equivalent functionality when response crosses jurisdictions or sectors

Note: Mechanism definitions are provided in Supplementary Material S3. Each documented-gap observation was assigned to one primary recurrent gap mechanism in the “Primary recurrent gap mechanism” field in Supplementary Material S4. Assignments are descriptive, with one primary mechanism per observation, and do not quantify the prevalence of system deficits.

**Table 4:** Descriptive source-stream comparison of documented preparedness–readiness gaps in PHEPA-derived and non-PHEPA evidence.

Characteristic	PHEPA-Derived	Non-PHEPA
Analytical observations (P01 interim synthesis excluded)	100	75
Observations with documented gaps	55	7
Observations with documented gaps, %	55.0%	9.3%
Readiness domains containing ≥ 1 documented gap	8/8	4/8
Formalisation and activation	11	0
Interoperability and visibility	14	3
Scalability and sustainability	13	3
Exercise and event evidence	3	1
Learning and corrective-action closure	5	0
Cross-sector and cross-border integration	9	0

Note: PHEPA-derived observations are from individual country PHEPA reports; the cross-country PHEPA interim synthesis was excluded from numerical estimates because its findings overlap with individual country reports. Mechanism counts were calculated from the primary recurrent gap-mechanism field in Supplementary Material S4. Each documented gap has one primary assignment; counts are descriptive and were not subjected to statistical testing.

**Table 5:** Leave-one-country sensitivity analysis for high-contribution PHEPA reports.

Analytical Sample	Observations, *n*	Documented Gaps, *n* (%)	Readiness Domains Containing ≥ 1 Documented Gap
Full quantitative sample	175	62 (35.4%)	8/8
Excluding Cyprus	148	44 (29.7%)	7/8
Excluding Cyprus and Liechtenstein	136	35 (25.7%)	7/8

Note: Cyprus contributed 27 observations and 18 documented gaps; Liechtenstein contributed 12 observations and 9 documented gaps. When Cyprus was excluded, D4 no longer contained a documented gap, but interoperability and visibility (12 observations) and scalability and sustainability (14 observations) remained the most frequent recurrent mechanisms. With Cyprus and Liechtenstein excluded, the same two mechanisms remained most frequent (10 and 11 observations, respectively). The underlying source-level counts are reported in the Source_Contribution_Summary worksheet of Supplementary Material S4.

## Data Availability

The data presented in this study were derived from publicly available policy documents and open-access institutional datasets. All sources are cited within the article.

## Supplementary Materials

The following supporting information can be downloaded at: https://www.mdpi.com/article/10.3390/healthcare14203529/s1, Supplementary Material S1: Master Documentary Corpus; Supplementary Material S2: Institutional Search Strategy and Document Identification; Supplementary Material S3: EU/EEA Operational Health Readiness Codebook; and Supplementary Material S4: Full Extraction and Coding Matrix (XLSX).

## Abbreviations

The following abbreviations are used in this manuscript:

AAR	After-Action Review
AI	Artificial Intelligence
CBRN	Chemical, Biological, Radiological and Nuclear
COVID-19	Coronavirus Disease 2019
ECDC	European Centre for Disease Prevention and Control
EEA	European Economic Area
EIOS	Epidemic Intelligence from Open Sources
ENISA	European Union Agency for Cybersecurity
EU	European Union
EUHTF	EU Health Task Force
HEPR	Health Emergency Preparedness, Response and Resilience
HERA	Health Emergency Preparedness and Response Authority
IAR	In-Action Review
IHR	International Health Regulations
IMS	Incident Management System
MCM	Medical Countermeasure
MPHS	Medical and Public Health Services
NATO	North Atlantic Treaty Organization
PHEOC	Public Health Emergency Operations Centre
PHEPA	Public Health Emergency Preparedness Assessment
RCCE	Risk Communication and Community Engagement
RCCE-IM	Risk Communication, Community Engagement and Infodemic Management
SOP	Standard Operating Procedure
WHO	World Health Organization
