# Supplementary material for: From Preparedness to Documented Operational Readiness: Mapping Public Evidence Across the EU/EEA Health-Security Architecture
Source: Healthcare (Basel). 2026 Oct 20;14(20):3529. doi: 10.3390/healthcare14203529 (PMC13704503; doi:10.3390/healthcare14203529)
Supplement: Supplementary file 1 [file healthcare-14-03529-s001.zip › Supplementary Material S1. Master Documentary Corpus.pdf]

Supplementary Material S1. Master Documentary Corpus

From Preparedness to Documented Operational Readiness: Mapping Public Evidence across the EU/EEA Health-Security Architecture

Purpose. This supplementary material provides the complete documentary corpus used in the study. Primary analytic sources contributed directly to the coded analysis. Supporting/contextual sources were retained for methodological or implementation triangulation but were not counted among the primary analytic sources. Foundational and benchmark documents informed the legal or conceptual framework but were not treated as evidence of contemporary EU/EEA operational readiness. Threat-context terms are retained to describe how sources frame potential threats; they are not treated as configuration-specific evidence of readiness for hybrid, compound or cascading crises.

Corpus summary. The inventory contains 45 documents: 35 primary analytic sources, 3 supporting/contextual sources, and 7 foundational or benchmark documents. Distinct documents or webpages providing substantive analytical evidence were assigned separate source IDs; locator or access pages were not counted separately. Document IDs correspond to the full extraction and coding matrix. The matrix separately flags document–capability observations and source-level threat-framing references.

Table S1A. Primary analytic sources - EU-level policy, implementation, operational, exercise and deployment evidence (n = 22)

ID	Institution / source	Publication date / year	Document title	Geographic scope	Primary domain(s)	Analytical role	Source	Notes / source handling
**EU01**	European Commission	2025-03-26	EU Preparedness Union Strategy	EU	D1,D2,D5,D6,D8	Strategic architecture	Source link	Use Communication/Annex, not factsheet as primary evidence.
**EU02**	European Commission / DG SANTE	2025-11-28	Commission Communication introducing the Union prevention, preparedness and response plan for health crises	EU/EEA	D1,D2,D3,D4,D5,D6, D7,D8	Health-crisis governance and operational architecture	Source link	Communication plus underlying Union Plan/SWD.
**EU03**	European Commission / DG SANTE	2025-11-28	Union prevention, preparedness and response plan for health crises (SWD / operational plan)	EU/EEA	D1,D2,D3,D4,D5,D6, D7,D8	Operational toolbox and activation/coordination evidence	Source link	Treat separately from the short introducing Communication.
**EU04**	European Commission	2025-07-09	EU stockpiling strategy: Boosting the EU's material preparedness for crises (COM(2025) 528)	EU	D2,D4,D5,D7,D8	Stockpiling, logistics, dependencies, cascading risks	Source link	Includes annex on future rescEU stockpiles.
**EU05**	European Commission / HERA	2025-07-09	Preparing the EU for the next health crisis: A Medical Countermeasures Strategy (COM(2025) 529)	EU	D1,D2,D5,D8	MCM threat prioritisation, manufacturing, procurement, stockpiling, rapid deployment	Source link	Analyse Communication and annexes as one policy package.
**EU06**	HERA	2025-04-09	HERA 2025 Work Plan	EU	D1,D2,D5,D8	Implementation planning and funded preparedness actions	Source link	Annual work programme.
**EU07**	HERA	2026-06-23	HERA 2026 Work Plan	EU	D1,D2,D5,D8	Implementation planning; threat intelligence and MCM operationalisation	Source link	Includes ATHINA/EIOS, wastewater surveillance, threat prioritisation.
**EU09**	HERA	2026-01-09	Comprehensive Health Threat Prioritisation Assessment for Medical Countermeasures	EU	D1,D5	Threat prioritisation linked to MCM preparedness	Source link	Use underlying assessment if available; news page is locator.
**EU10**	European Commission	2025-03-11	Proposal for a Critical Medicines Act (COM(2025) 102)	EU/EEA	D5	Security of supply / manufacturing resilience	Source link	Policy-level evidence; proposal, not enacted mechanism.
**EU11**	European Commission	2025-01-15	European action plan on the cybersecurity of hospitals and healthcare providers	EU	D7,D8	Health-sector cyber preparedness, detection, response and recovery	Source link	Sector-specific cyber architecture.
**EU12**	ENISA	2026-07-22	First steps forward for the implementation of the Health Action Plan	EU	D7	Implementation evidence for health cybersecurity action plan	Source link	Includes updated procurement guidance and Support Centre service-catalogue work.
**EU13**	European Commission	2026-07-07	EU Action Plan on Cybersecurity and Artificial Intelligence	EU	D7	AI as capability-enabler and threat-amplifier	Source link	Cross-cutting AI modifier source.
**EU14**	ECDC	2025-11-21	EU Health Task Force marks first 1,000 days of operations	EU/EEA	D2,D3	Real-world deployable workforce evidence	Source link	Documents 36 requests and 33 assignments since May 2023.
**EU15**	DG ECHO / UCPM	2026-09-12	rescEU - operational capacities status	EU/UCPM	D4,D5	Operational reserve, MedEvac, EMT and CBRN capacity evidence	Source link	Living webpage; record access date. States 17 of 22 developing stockpiles fully operational and MedEvac used for Ukraine.
**EU16**	ECDC / Europol	2025-11-19	Operation Vector: cross-sectoral simulation exercise on deliberate biological threats	EU/EEA	D1,D2,D6,D8	Exercise evidence for explicitly tested public-health functions in a deliberate biological scenario	Source link	Completed exercise; coded to each explicitly tested capability. Deliberate biological scenario alone is not hybrid-scenario evidence.
**EU17**	ECDC	2026-03-31	Belgium simulation exercise on viral haemorrhagic fever outbreak	National/EU support	D1,D2,D6,D8	Exercise evidence for surveillance, coordination and risk communication	Source link	Completed exercise; coded to each explicitly tested capability. No clinical-surge code was inferred from isolation-unit discussion.
**EU18**	ECDC	2026-02-04	Malta simulation exercise on vector-borne disease outbreak	National/EU support	D2,D8	Exercise evidence for cross-sector outbreak coordination	Source link	Completed exercise; coded only to explicitly tested capabilities.
**EU19**	European Commission / DG HOME	2026-07-10	Guidance to strengthen resilience of critical infrastructure	EU	D2,D4,D7,D8	Cross-sector dependencies, public-health emergencies, hybrid threats and recovery	Source link	Use underlying Communication when available.
**EU20**	ECDC	2026-04-10	Epi+ pilot phase — going live June 2027	EU/EEA	D1,D2,D7	Digital event-based surveillance platform and documented interoperability gap	Source link	Discovered in update search; pre-operational platform, testing begins Sep 2026.

ID	Institution / source	Publication date / year	Document title	Geographic scope	Primary domain(s)	Analytical role	Source	Notes / source handling
**EU21**	ECDC	2026	EU Health Task Force Bulletin 2025	EU/EEA	D2,D3,D8	Operational workforce deployments, preparedness support and expert-pool maturity	Source link	Adds 2025 operational activity and workforce-pool status.
**EU22**	European Commission	2026-06-05	Cybersecurity of hospitals and healthcare providers	EU	D7	Cybersecurity implementation roadmap and planned services	Source link	Separate dated fact page underlying OBS063; the proposed subscription and response services support P, not M.
**EU23**	European Commission / DG ECHO	2026-04-16	5,000 patients medically evacuated from Ukraine for urgent treatment	EU/UCPM	D4	Documented real-world cross-border medical evacuation	Source link	Separate dated news item underlying OBS060; not a locator for the dynamic rescEU capacity page (EU15).

Table S1B. Primary analytic sources - ECDC preparedness-assessment evidence (n = 13)

ID	Institution / source	Publication date / year	Document title	Geographic scope	Primary domain(s)	Analytical role	Source	Notes / source handling
**P01**	ECDC	2026-06-04	Preliminary findings from the first cycle of Public Health Emergency Preparedness Assessments – Interim report	15 EU/EEA countries	D1,D2,D3,D4,D5,D6, D7,D8	Main cross-country implementation/gap synthesis	Source link	Contextual cross-country source. Its 15 observations, including 12 documented gaps, are retained in the full matrix but excluded from quantitative estimates to avoid overlap with individual country reports.
**P04**	ECDC	2024-07-11	PHEPA Belgium, 2024	Belgium	Multiple	Country implementation evidence	Source link	Five capacities assessed in depth; remaining Article 7 capacities validated.
**P05**	ECDC	2026-06-25	PHEPA Cyprus, 2025	Cyprus	Multiple	Country implementation evidence	Source link	Country visit Dec 2025.
**P06**	ECDC	2025-04-09	PHEPA Estonia, 2024	Estonia	Multiple	Country implementation evidence	Source link	Contains multi-hazard plans, IMS, supply chain and workforce findings.
**P07**	ECDC	2025-12-08	PHEPA Finland, 2024	Finland	Multiple	Country implementation evidence	Source link	Country visit June 2024.
**P08**	ECDC	2026-01-27	PHEPA France, 2025	France	Multiple	Country implementation evidence	Source link	Country-level operational and exercise evidence.
**P09**	ECDC	2025-10-13	PHEPA Iceland, 2025	Iceland	Multiple	Country implementation evidence	Source link	All-hazards/civil-protection strengths and workforce/formalisation gaps.
**P10**	ECDC	2025-10-10	PHEPA Latvia, 2025	Latvia	Multiple	Country implementation evidence	Source link	Country-level readiness evidence.
**P11**	ECDC	2026-09-09	PHEPA Liechtenstein, 2026	Liechtenstein	Multiple	Country implementation evidence	Source link	Published immediately before search closure; the source link opens the specific ECDC report page, and the matrix links the report PDF.
**P12**	ECDC	2026-06-16	PHEPA Lithuania, 2025	Lithuania	Multiple	Country implementation evidence	Source link	Country visit Sep 2025.
**P13**	ECDC	2025-07-02	PHEPA Luxembourg, 2024	Luxembourg	Multiple	Country implementation evidence	Source link	Useful for interoperability/workforce/scaling gaps.
**P14**	WHO Europe / ECDC	2025-08-26	Joint External Evaluation and PHEPA mission report: Netherlands, 2025	Netherlands	Multiple	Country implementation evidence	Source link	Joint WHO/ECDC assessment; mission 27–31 Jan 2025.
**P15**	ECDC	2025-07-24	PHEPA Spain, 2024	Spain	Multiple	Country implementation evidence	Source link	Includes RCCE/infodemic capacity in-depth.

Table S1C. Supporting/contextual sources (n = 3)

ID	Institution / source	Publication date / year	Document title	Geographic scope	Primary domain(s)	Analytical role	Source	Notes / source handling
**EU08**	HERA	2026-06-16	Annual activity report 2025 - HERA	EU	D1,D2,D5,D8	HERA implementation cross-check; retained as supporting evidence because primary coded HERA work-plan and operational sources cover the relevant capabilities.	Source link	Prefer for evidence of completed actions over work plans.
**P02**	ECDC	2026-02-18	Guide for countries undergoing an ECDC Public Health Emergency Preparedness Assessment	EU/EEA	All	PHEPA assessment-method guide used to interpret scope, comparability and assessment procedures; not coded as an implementation outcome source.	Source link	Used to interpret PHEPA scope and comparability.
**P03**	European Commission / ECDC	2026-03-25	Overview of recommendations on preparedness and response to serious cross-border threats to health	EU/EEA	D1,D2,D3,D4,D5	Cross-country recommendations derived largely from the 2023 reporting cycle; retained for contextual triangulation rather than current-period implementation coding.	Source link	Supplementary implementation layer.

Table S1D. Foundational and external benchmark documents (n = 7)

ID	Institution / source	Publication date / year	Document title	Geographic scope	Primary domain(s)	Analytical role	Source	Notes / source handling
**F01**	European Parliament / Council	2022-11-23	Regulation (EU) 2022/2371 on serious cross-border threats to health	EU/EEA	All	Legal foundation	Source link	Outside primary time window; retained as foundational legal source.
**F02**	European Parliament / Council	2022-12-14	Directive (EU) 2022/2557 on the resilience of critical entities	EU/EEA	D2,D4,D7,D8	Critical-infrastructure legal foundation	Source link	Health is within essential-service resilience framework; includes public health emergencies and hybrid threats.
**F03**	European Parliament / Council	2022-12-14	Directive (EU) 2022/2555 (NIS2)	EU/EEA	D7	Cyber legal foundation	Source link	Outside primary time window; retained to contextualise health cybersecurity obligations.
**B01**	World Health Organization	2023-05-23	Health Emergency Preparedness, Response and Resilience (HEPR) architecture / collaborative surveillance framework	Global	All	Conceptual benchmark	Source link	External benchmark only; not evidence of EU implementation.
**B02**	World Health Organization	2026-03-23	Framework for health emergency preparedness and response capabilities for national public health agencies	Global	All	Capability benchmark	Source link	External benchmark used to refine domains/enablers.
**B03**	NATO	2026-09-15	2026 Resilience Baseline Requirements	Euro-Atlantic	D2,D3,D4,D5,D7,D8	Hybrid/civil-military resilience benchmark	Source link	External benchmark only; retained for conceptual context. It was not used to select the corpus, assign codes or calculate results.
**B04**	English et al. / BMJ Global Health	2024-08-29	Defining and identifying the critical elements of operational readiness for public health emergency events: a rapid scoping review	Global	Conceptual	Operational-readiness definition	Source link	Methodological/conceptual triangulation.

Notes. Primary analytic sources contributed directly to document–capability coding and synthesis. Supporting/contextual sources were retained for methodological interpretation or triangulation but were not counted among the 35 primary analytic sources. Foundational and benchmark documents were used to establish the legal or conceptual basis of the framework and were not treated as evidence of contemporary EU/EEA operational readiness. Domain assignments indicate the principal areas for which each source was considered relevant and do not imply that every document was coded to every listed domain. “Multiple” indicates that several operational-readiness domains were relevant; “All” indicates broad framework-level coverage. Terms such as “hybrid”, “compound” and “cascading” were treated as source framing unless the strict configuration-specific threshold in Supplementary Material 3 was met. For some reports, the inventory links the official publication page while Supplementary Material 4 records a direct report PDF; these are pointers to the same source, not separate documents.
