# Supplementary material for: From Preparedness to Documented Operational Readiness: Mapping Public Evidence Across the EU/EEA Health-Security Architecture
Source: Healthcare (Basel). 2026 Oct 20;14(20):3529. doi: 10.3390/healthcare14203529 (PMC13704503; doi:10.3390/healthcare14203529)
Supplement: Supplementary file 1 [file healthcare-14-03529-s001.zip › Supplementary Material S2. Institutional Search Strategy and Document Identification.pdf]

Supplementary Material S2. Institutional Search Strategy and Document Identification

From Preparedness to Documented Operational Readiness: Mapping Public Evidence across the EU/EEA Health-Security Architecture

Purpose. This supplementary material documents the purposive, structured institutional search used to identify policy, legal, implementation, assessment, exercise and operational sources for the study. Search terminology and combinations were adapted to each institution’s mandate, repository structure and vocabulary; a single identical search string was not applied across repositories.

Search scope. The primary analytical period was 1 January 2024 to 17 September 2026. Earlier documents were retained only when they provided an essential legal or conceptual foundation for the contemporary EU/EEA health-security architecture. The iterative analytical search closed on 17 September 2026; no formal saturation criterion was specified or assessed. Searches were supplemented by targeted domain-restricted web searches. Peer-reviewed literature was used selectively for conceptual and methodological interpretation and was not treated as a separately searched primary analytical corpus.

Codebook timing and audit. The institutional search closed on 17 September 2026; core domains and P/M/O/E/R rules were finalised after pilot testing on 18 September and used for the original extraction. Subsequent, non-preregistered revisions are recorded in Supplementary Material 3: Version 1.1 (8 October) clarified threat-context thresholds and NR; Version 1.2 (9 October) clarified that E means completed testing and renamed one gap mechanism without changing counts from Version 1.1; Version 1.3 (12 October) audited source identity, intent and interaction for all 190 observations, assigned separate IDs to two substantive dated webpages, and corrected one M code; Version 1.4 (15 October) removed unsupported O codes from OBS064 and OBS086 and an R code from OBS166, then recalculated evidence totals and Figure 1; Version 1.5 (15 October) made observation accounting and the whole-source exclusion of P01 explicit without changing codes or results; and Version 1.6 (15 October) aligned the permitted interaction values with the matrix and recorded row-level rationales for multi-hazard and boundary cases without changing classifications. These revisions did not reopen the primary search. Original screening and coding and the later consistency checks were undertaken by one researcher; no independent duplicate coding or agreement statistic is reported. The 2026 NATO Resilience Baseline Requirements were used only as an external benchmark.

Table S2A. Institutional and repository search strategy

Institution / repository	Institutional focus	Core search concepts	Representative combinations	Document types targeted	Analytical role
European Commission / DG SANTE / HERA	EU health-security policy, preparedness architecture, medical countermeasures and implementation	operational readiness; health emergency preparedness; health security; medical countermeasures; stockpiling; manufacturing; threat assessment; deployment	“operational readiness” + health; “health emergency preparedness” + EU; “medical countermeasures” + stockpil*; manufacturing + emergency; deployment + health	Commission communications; strategies; preparedness/response plans; HERA work programmes; implementation and operational reports	Primary EU-level policy, mechanism and implementation evidence
ECDC	Preparedness assessment, surveillance, laboratories, deployable public-health expertise, exercises and learning	PHEPA; preparedness assessment; surveillance; laboratory surge; EU Health Task Force; exercise*; after-action; deployment	PHEPA + preparedness; surveillance + preparedness; laboratory + surge; “EU Health Task Force” + deployment; exercise* + public health	PHEPA country reports and interim synthesis; technical guidance; EUHTF reports; exercise/AAR material; operational notices	Principal national implementation evidence and selected EU-level operational/exercise evidence
European Medicines Agency (EMA)	Medicines availability, shortages and emergency-related pharmaceutical coordination	emergency preparedness; medicines shortages; critical medicines; medical countermeasures; supply continuity	emergency + medicines; shortages + preparedness; critical medicines + supply	Preparedness, shortage-management and implementation documents relevant to emergency pharmaceutical continuity	Targeted supporting/primary evidence where operational-readiness criteria were met
DG ECHO / Union Civil Protection Mechanism / rescEU	Civil-protection capacities, strategic reserves, logistics, medical evacuation and cross-border deployment	rescEU; stockpile*; medical evacuation; patient transfer; deployment; logistics; CBRN; medical reserve	rescEU + medical; stockpile* + deployment; “medical evacuation” + patient; CBRN + reserve; logistics + emergency	Capacity descriptions; deployment reports; operational notices; civil-protection implementation material	Primary evidence for cross-border scaling, stockpiles, logistics and real-world deployment
ENISA	Cybersecurity of hospitals, healthcare providers and health-sector digital resilience	health cybersecurity; hospital cybersecurity; cyber resilience; incident response; recovery; healthcare	cyber* + health; cybersecurity + hospital*; healthcare + incident response; cyber resilience + healthcare	Implementation guidance; sector reports; preparedness and response material	Primary/supporting evidence for digital and cyber-readiness mechanisms
EEAS / Council of the European Union	Hybrid threats, resilience, cross-sector coordination and civil–military/security interfaces	hybrid threat*; resilience; civil-military; information manipulation; deliberate threat; crisis coordination	“hybrid threat*” + health; civil-military + preparedness; information manipulation + crisis; resilience + critical infrastructure	Council/EEAS policy pages, conclusions, strategic and explanatory documents	Policy and contextual evidence for deliberate/hybrid threat framing and cross-sector interfaces
EUR-Lex	Binding EU legal instruments and formal Commission/Council documents	serious cross-border threats to health; preparedness; critical entities; cybersecurity; health emergency; resilience	“serious cross-border threats to health”; preparedness + regulation; critical entities + resilience; health + cybersecurity	Regulations; directives; decisions; Commission communications and other formal EU instruments	Foundational legal evidence and formal policy architecture
World Health Organization (WHO)	External conceptual and technical preparedness/readiness frameworks	HEPR; operational readiness; health emergency capabilities; all-hazards; collaborative surveillance; scalable care	HEPR + readiness; “health emergency preparedness” + capabilities; all-hazards + health emergency	Frameworks; technical guidance; conceptual documents	External conceptual/technical benchmark only; not evidence of EU/EEA implementation
NATO	External resilience benchmark for civil–military, continuity, mass-casualty and disruptive-crisis dimensions	resilience baseline requirements; civil-military; health system continuity; mass casualties; medical stockpiling; disruptive health crises	resilience + health; civil-military + medical; healthcare capacity + resilience; medical stockpiling + exercise	Official resilience requirements and related technical/strategic documents	External benchmark only; not an analytical unit

Table S2B. Cross-cutting search concept clusters

Concept cluster	Representative terms	Primary evidence sought
Readiness and preparedness	“operational readiness”; “health emergency preparedness”; “health security”; preparedness; response	Definitions, preparedness architecture, implementation mechanisms and readiness functions
Surge and continuity	“surge capacity”; workforce; “continuity of essential health services”; redundancy; patient transfer; evacuation	Mobilisation, scaling, continuity, redistribution and cross-border care capacity
Medical countermeasures and supply chains	“medical countermeasures”; stockpil*; procurement; manufacturing; logistics; deployment	Strategic reserves, inventory/needs visibility, scalable production/procurement and deployment
Coordination, deliberate and hybrid threats	“emergency coordination”; “civil-military”; “hybrid threat*”; deliberate; CBRN; information manipulation	Activation, cross-sector/cross-border coordination, civil–military mechanisms and deliberate-threat preparedness
Digital, cyber and critical infrastructure	cyber*; interoperability; digital continuity; “critical infrastructure”; hospitals; healthcare	Data interoperability, cyber prevention/response and dependencies affecting continuity
Exercises, operational use and learning	exercise*; simulation; deployment; activation; after-action; corrective action	Completed exercises, real-world activation/deployment and evidence of learning/adaptation
Compound and cascading disruption	“compound crisis”; cascading; multi-hazard; interdependence; simultaneous disruption	Explicit documentary evidence of a compound configuration, causal cascading chain or coordinated hybrid campaign; general references to interdependence, critical infrastructure or threat labels were retained only as threat framing.

Document-identification procedure. Searches were conducted iteratively within each institutional source using the concept clusters above. Candidate documents were assessed for relevance to at least one predefined operational-readiness capability and for substantive evidence on policy commitment, established mechanisms, activation or scaling, exercises, real-world use, implementation status, dependencies or documented gaps. Threat terminology alone was not treated as evidence of configuration-specific readiness. Duplicates and superseded versions were excluded where a final version was available. Material unrelated to health emergency readiness, routine health-system items and commentary without substantive evidence was excluded; speeches, promotional content and press releases were generally not retained when an underlying source was available. Official event or deployment notices were retained when they represented the authoritative public record of a completed exercise or real-world activation. The final included and contextual documents are listed in Supplementary Material 1.

Notes. This was a purposive institutional search rather than a systematic review. Individual query dates and a candidate/exclusion log were not retained, so identified, screened or excluded counts by reason cannot be reconstructed. Table S2C reports 45 retained sources without a PRISMA-like flow. Search combinations are illustrative; codebook history appears in Supplementary Material 3.

Table S2C. Retained-source disposition and limitation of retrospective screening reconstruction.

Record category	n	Treatment in the revised study
Primary analytic sources	35	Retained in the primary corpus.
Sources contributing to quantitative estimates	34	Included in the 175-observation frequency analysis.
PHEPA interim synthesis (P01)	1	Retained contextually in the full matrix; 15 observations, including 12 documented gaps, excluded from numerical estimates because the source overlaps with individual country reports.
Supporting/contextual sources	3	Retained for methodological or implementation triangulation; not coded as primary analytic evidence.
Foundational/benchmark sources	7	Used for legal or conceptual context only; not primary analytic evidence.
Total retained documentary sources	45	Complete retained inventory reported in Supplementary Material 1.
Candidate and excluded records	Not reconstructable	No contemporaneous candidate/exclusion log was retained; no estimated count is supplied.
