# Supplementary material for: From Preparedness to Documented Operational Readiness: Mapping Public Evidence Across the EU/EEA Health-Security Architecture
Source: Healthcare (Basel). 2026 Oct 20;14(20):3529. doi: 10.3390/healthcare14203529 (PMC13704503; doi:10.3390/healthcare14203529)
Supplement: Supplementary file 1 [file healthcare-14-03529-s001.zip › Supplementary Material S3. EU-EEA Operational Health Readiness Codebook.pdf]

Supplementary Material S3. EU/EEA Operational Health Readiness Codebook

Version 1.6 - Threat-context consistency check (15 October 2026)

**Study scope**	European Union / European Economic Area (EU/EEA)
**Primary analytical period**	1 January 2024 – 17 September 2026
**Primary unit of analysis**	Document–capability observation
**Analytical purpose**	Map public evidence of operationalisation across the EU/EEA health-security architecture and describe how included documents frame complex threats.

Version history. The core operational domains and P/M/O/E/R evidence framework used for original extraction were finalised after pilot testing on 18 September 2026. Version 1.1 (8 October 2026) clarified threat-context thresholds and NR treatment retrospectively; these decisions were not preregistered. Version 1.2 (9 October 2026) clarified that E records completed testing without implying adequate performance and renamed the exercise and event evidence gap mechanism; no numerical result changed from Version 1.1 to Version 1.2. Version 1.3 (12 October 2026) records a retrospective matrix audit of source identity, intent and interaction, including one corrected M code. Version 1.4 (15 October 2026) records a targeted audit of laboratory/surveillance, cyber and medical-evacuation sources: O was removed from OBS064 and OBS086, and R from OBS166. Counts and Figure 1 were updated; the definitions were unchanged. Version 1.5 (15 October 2026) documents observation counts, the whole-source P01 exclusion and the gap denominator without altering any row assignment, evidence code or result. Version 1.6 (15 October 2026) harmonised the interaction value list with the actual matrix, clarified how threat-framing notes accompany capability observations, and recorded a whole-matrix value check plus reasons for all 19 multi-hazard rows and seven boundary cases. No intent, interaction, evidence or gap assignment changed, and the numerical results were unchanged. These revisions are retrospective, not independent duplicate coding.

1. Purpose and coding principles

- The codebook maps publicly available documentary evidence of operationalisation across the EU/EEA health-security architecture using a capability-based Framework Method.
- Capabilities are coded only when supported by explicit documentary evidence; analytical inference and source-level threat framing are recorded separately from capability evidence.
- The codebook distinguishes policy architecture from established mechanisms, operational functionality, completed exercise testing and real-world use.
- Absence of public evidence is never interpreted as evidence that a capability is absent.
- Country-level PHEPA evidence is used for implementation patterns, not for country ranking or league-table comparisons.
- Threat-context coding characterises how an included document frames a threat. It does not assess readiness for hybrid, compound or cascading crisis configurations.

2. Operational readiness domains

Eight operational domains are used. Legal authority, flexible emergency financing, and evidence-to-policy mechanisms are treated as cross-cutting enablers rather than stand-alone operational domains.

D1. Threat intelligence, surveillance & diagnostics

Code	Indicator	Operational definition
**D1.1**	Integrated surveillance	Mechanisms that collect, integrate, analyse and share health or threat data across relevant surveillance streams and sectors.
**D1.2**	Laboratory & diagnostic surge	Documented ability to expand diagnostic, sequencing, specialist laboratory or reference-testing capacity during an emergency.
**D1.3**	Risk assessment & foresight	Structured processes for anticipating, characterising or assessing emerging, deliberate, multi-hazard, compound or cascading threats.
**D1.4**	Situational awareness	Mechanisms that generate and distribute timely, actionable information on evolving threats, impacts, capacities and response needs.

D2. Governance, activation & emergency coordination

Code	Indicator	Operational definition
**D2.1**	Activation mechanisms	Defined triggers, thresholds, procedures or legal/administrative arrangements for activating emergency response structures or measures.
**D2.2**	Emergency coordination	Formal command, coordination, crisis-management or incident-management structures with defined roles and responsibilities.
**D2.3**	Cross-border / cross-sector coordination	Arrangements linking countries, agencies or sectors for coordinated assessment, decision-making, resource use or response.
**D2.4**	Civil–military coordination	Formal health-related arrangements for civilian–military planning, information exchange, logistics, medical support, evacuation or resource coordination.

D3. Workforce & surge mobilisation

Code	Indicator	Operational definition
**D3.1**	Surge workforce	Reserve, deployable or expandable workforce arrangements capable of increasing staffing during an emergency.
**D3.2**	Rapid mobilisation	Procedures for recall, credentialling, redistribution, deployment or cross-border mobilisation of personnel.
**D3.3**	Training & competencies	Defined emergency preparedness competencies and structured training relevant to operational roles.
**D3.4**	Workforce continuity	Arrangements designed to maintain workforce availability during prolonged disruption, including replacement, occupational protection and fatigue/absence management.

D4. Scalable care & continuity of essential health services

Code	Indicator	Operational definition
**D4.1**	Clinical surge capacity	Ability to expand acute, emergency, critical-care, prehospital or other clinically necessary capacity in response to increased demand.
**D4.2**	Patient redistribution / evacuation	Mechanisms for interfacility, interregional or cross-border transfer, evacuation, redistribution or medical transport of patients.
**D4.3**	Essential-service continuity	Formal arrangements to sustain essential health services while emergency demand or disruption is ongoing.
**D4.4**	Infrastructure redundancy	Backup or alternative facilities, utilities, power, communications, digital systems, supply arrangements or alternative care pathways supporting continuity.

D5. Medical countermeasures, stockpiles & supply chains

Code	Indicator	Operational definition
**D5.1**	Strategic reserves	Established stockpiles or reserves of medicines, vaccines, diagnostics, PPE, equipment, blood products or CBRN-related countermeasures.
**D5.2**	Inventory & needs visibility	Mechanisms that provide information on stock levels, shortages, duplication, consumption, projected demand or priority needs.
**D5.3**	Procurement / manufacturing surge	Joint procurement, advance contracts, reserved manufacturing, scalable production or other mechanisms intended to increase supply rapidly.

Code	Indicator	Operational definition
**D5.4**	Logistics & deployment	Mechanisms for release, transport, distribution, cross-border movement and rapid delivery of critical medical resources.

D6. Community protection & information integrity

Code	Indicator	Operational definition
**D6.1**	Risk communication	Structured communication of risk, protective measures and crisis information to populations, professionals or stakeholders.
**D6.2**	Community engagement	Mechanisms that involve communities, civil society, professional groups or other stakeholders in preparedness, response or adaptation.
**D6.3**	Population protection	Organised public-health and social measures or other interventions intended to reduce population exposure, transmission or harm.
**D6.4**	Information integrity	Mechanisms to monitor, anticipate or respond to misinformation, disinformation, information manipulation or other threats to trusted crisis communication.

D7. Digital, cyber resilience & interoperability

Code	Indicator	Operational definition
**D7.1**	Data interoperability	Ability to exchange, integrate and use relevant health and emergency data securely across organisations, jurisdictions or information systems.
**D7.2**	Cyber prevention & detection	Health-sector-specific measures for cyber risk reduction, monitoring, early warning, detection and preparedness.
**D7.3**	Cyber response & recovery	Incident-response, backup, restoration, recovery and continuity mechanisms following cyber disruption.
**D7.4**	Digital / communication continuity	Redundant and resilient digital, telecommunications or crisis-communication infrastructure capable of functioning during disruption.

D8. Exercises, learning, recovery & adaptation

Code	Indicator	Operational definition
**D8.1**	Exercise programme	Regular tabletop, simulation, functional, full-scale or other structured exercises used to test preparedness and response.
**D8.2**	Compound / hybrid scenario testing	Completed exercises that explicitly test either: (i) two or more concurrent hazards or pressures interacting to affect the same capability, population or service; (ii) a primary disruption triggering secondary failures in dependent systems that affect health-system functioning; or (iii) a coordinated hybrid campaign involving malign intent and combined instruments. An intentional biological, CBRN, cyber or misinformation scenario alone is not sufficient.
**D8.3**	After-action learning	Structured after-action reviews, intra-action reviews, lessons-identified processes or equivalent evaluations following exercises or real events.
**D8.4**	Corrective action & adaptation	Documented evidence that identified gaps or lessons led to changes in plans, structures, training, resources or operational procedures.

3. Evidence-of-operationalisation coding

Evidence codes are non-mutually exclusive and are not converted into a numerical readiness score. Multiple codes may be assigned to the same document–capability observation when supported by evidence.

Code	Label	Assignment rule	Do not assign when…
**P**	Policy	A formal strategy, plan, law, requirement or explicit policy commitment exists.	The source only expresses a general aspiration, commentary or non-authoritative recommendation.
**M**	Mechanism	A named structure, procedure, resource, stockpile, workforce arrangement, coordination mechanism or implementation instrument has been established.	The mechanism is merely proposed, planned or announced for future creation.
**O**	Operational	The source explicitly documents functionality for activation, mobilisation, deployment, redistribution, release or scaling in an emergency.	The source simply labels a mechanism "operational" without describing actionable functionality.
**E**	Exercised	The specified capability was tested in a completed tabletop, simulation, functional, full-scale or comparable exercise. Completion of testing does not establish adequate performance or achievement of objectives.	An exercise is planned, recommended or scheduled but not documented as completed.
**R**	Real-world use	The capability was activated or deployed during a real emergency, incident, crisis or operational response.	The evidence relates only to an exercise, pilot or hypothetical scenario.

3.1. Key decision rules

- Codes are parallel and non-ordinal. An observation may be coded P+M+O+E+R when each attribute is supported; co-occurrence does not indicate development along a scale or a mandatory sequence.
- A stockpile that physically exists is M. It becomes O only when release/deployment/scaling functionality is documented.
- A future initiative announced in a strategy is P, not M, until establishment is documented.
- E requires direct documentation that the specified capability was tested in a completed exercise; it records completion of testing, not whether the capability performed adequately or met exercise objectives. Explicit exercise shortcomings are captured separately as documented gaps. R requires an identified real-world event or deployment involving that capability.
- A claim that a system is "operational" does not automatically justify O; the source must describe actionable functionality.
- The revised analysis does not use NR as an evidence code. An explicitly reported shortcoming is recorded as a documented gap, with a "documented gap only" observation when no P/M/O/E/R evidence attribute is supported. Lack of public detail is recorded in a limitation or audit note and is never interpreted as capability absence.

3.2. Observation accounting and source overlap

A row is a source-linked, indicator-specific capability or explicit limitation. Distinct independently supported capabilities or separately stated gaps can produce separate rows; several evidence attributes and one gap may coexist in a row. The full matrix contains 190 unique IDs: 168 capability-evidence rows, 21 gap-only rows and one context-only threat-framing reference. All 15 rows from Source ID P01, the interim synthesis of 15 assessments, were retained for audit but removed as a block from frequency calculations because its aggregate findings could overlap individual country PHEPA reports. No one-to-one match between P01 and country-report passages was asserted. The remaining 175 rows comprise 155 capability-evidence, 19 gap-only and one threat-framing reference; 174 of these have a domain assignment.

For the gap tally, count a row once if Gap status is "Documented gap"; do not count absent P/M/O/E/R attributes as gaps. Each counted gap has one primary recurrent gap mechanism. The full matrix has 74 gap rows; 12 belong to P01, leaving 62/175 (35.4%) in the quantitative subset. The 62 comprise 43 rows also carrying capability evidence and 19 gap-only rows. The context-only reference remains in the general 175-row denominator and is omitted from the 174-row domain display. Supplementary Material 4 supplies source IDs, observation roles, gap flags and formula-backed reconciliation.

4. Threat-context classification

Threat context is coded on two independent axes: intent and interaction structure. This prevents "hybrid" from being used as a synonym for any complex emergency.

Field	Allowed values	Coding rule
**Intent**	Natural/accidental; deliberate; hybrid campaign; unclear	Assign hybrid campaign only where the source explicitly documents coordinated harmful activity, malign intent and combined instruments. Do not use it for an isolated reference to misinformation, cyber risk, AI dual use, CBRN or deliberate biological threat.
**Interaction structure**	Single hazard; multi-hazard; not specified/not applicable; compound crisis; cascading disruption	Single hazard requires one identified hazard class or event; sectors, functions or pathogens within one class do not multiply hazards. Multi-hazard requires two distinct hazard classes or an explicit all-/multi-hazard scope tied to the observation, without implying interaction. Use not specified/not applicable if no hazard configuration is identifiable. Compound requires concurrent hazards or pressures explicitly interacting to affect the same function, population or service. Cascading requires a stated initiating event causing secondary failures in dependent systems that affect health-system functioning. Generic interdependencies or continuity language are insufficient.
**AI role**	None; capability-enabling; threat-amplifying; both	AI is a modifier, not an operational readiness domain.
**Governance level**	EU; national; shared	Use the level at which the documented capability is governed or activated. Civil-military involvement is recorded separately as an analytical component.
**Cross-border scalability**	Documented; unclear; not publicly reported	Code only where cross-border mobilisation, transfer, deployment or shared use is explicitly described.
Threat-framing note	Free text	Record explicit hybrid, compound or cascading terminology, or a boundary case that could be mistaken for one of these configurations, where the strict threshold is not met. A note does not itself constitute configuration-specific readiness evidence or require a separate observation role.

When a source uses a hybrid, compound or cascading label without satisfying the relevant configuration threshold, record the wording in the Threat-framing note rather than assigning a configuration code. The note may accompany a capability-evidence row. Use the separate Threat-framing reference observation role only when the passage supplies context but no independently supported capability.

4.1. Consistency check and boundary cases

The retrospective source and threat audit in Version 1.3 reviewed all 190 matrix observations. Version 1.6 checked that every row has a permitted value on both threat axes: intent 40 natural/accidental, 15 deliberate and 135 unclear; interaction 49 single hazard, 19 multi-hazard and 122 not specified/not applicable. There were no hybrid-campaign, compound-crisis or cascading-disruption assignments. The Coding-decision note now gives a row-specific scope rationale for every multi-hazard assignment. The seven boundary cases are listed in the Threat_Context_Audit worksheet of Supplementary Material 4, with the corresponding full-matrix row IDs and source locators. This was a single-researcher consistency check, not independent recoding or an agreement assessment.

5. Cross-cutting enablers, dependencies and gaps

5.1. Cross-cutting enablers

- Legal authority / mandate
- Flexible emergency financing / resource mobilisation

- Evidence-to-policy / decision support

5.2. Dependencies

Dependencies are coded inductively where they materially enable or constrain operational readiness. Typical categories include:

- Workforce
- Energy and utilities
- Transport and logistics
- Digital infrastructure and telecommunications
- Laboratory capacity
- Manufacturing and procurement
- Supply chains
- Financing
- Inter-organisational / cross-sector interoperability
- Public trust and communication

5.3. Gap coding

Gap status	Rule
**Documented gap**	Assign only where the source explicitly identifies a shortcoming, limitation, insufficient capacity, implementation problem, bottleneck or corrective need.
**No gap reported**	The source does not report a gap; this does not demonstrate that no gap exists.
**Not assessed / evidence gap**	The issue was outside the source scope or no adequate public evidence was available. Keep separate from a documented capability gap.

6. Minimum extraction fields

- Document ID
- Institution / source
- Publication date
- Geographic level
- Exact page / section / location
- Verbatim or close-paraphrase supporting evidence
- Readiness domain
- Indicator code
- Evidence code(s): P/M/O/E/R, where evidence is present
- Observation role: capability evidence; documented gap only; or threat-framing reference
- Intent classification
- Interaction structure
- Governance level
- Cross-border scalability
- Civil–military component
- AI role
- Cross-cutting enabler(s)
- Critical dependency/dependencies
- Gap status
- Documented gap / corrective recommendation

7. Source hierarchy and evidence handling

- Use binding legislation and primary institutional documents to establish formal mandates and policy architecture.
- Use implementation, operational and assessment reports for claims about functioning, activation, scalability or implementation status.
- Require completed exercise documentation for E and real-world deployment/activation documentation for R.

- Use peer-reviewed literature for triangulation and interpretation; do not use it to override authoritative primary documentation of whether an EU/EEA mechanism formally exists.
- Use press releases primarily to locate underlying documentation. Retain an official event/deployment notice only when it is the authoritative public record of a completed exercise or real-world activation.
- For PHEPA country reports, do not infer absence of capability from silence because assessment depth varies by capacity and country.

8. Ambiguity resolution rules

Situation	Decision rule
**Two domains appear relevant**	Assign the observation to the domain representing the principal operational function; cross-code only when the same evidence independently supports two distinct capabilities.
**Policy and mechanism described together**	Assign both P and M if the source clearly documents that the mechanism has been established, not merely proposed.
**Exercise tests several capabilities**	Create separate document–capability observations for each capability actually tested.
**Real event mentioned without operational detail**	R may be assigned only to the specific capability whose activation/use is documented; do not generalise R to adjacent capabilities.
**Cross-border mechanism exists but national uptake is unclear**	Code the EU-level mechanism as supported; do not infer national implementation. Record "national uptake not reported" in an audit note rather than use an evidence code.
**Security-sensitive information is unavailable**	Record a disclosure limitation in an audit note; do not infer lack of readiness or create a capability-deficit observation from missing public detail.
**Hybrid intent is uncertain**	Use "unclear" rather than assuming hybrid intent from complexity alone.
Source refers to hybrid threat, cyber risk, misinformation or a deliberate incident only	Record a threat-framing note. Assign deliberate or unclear intent unless coordinated malign activity combining instruments is explicitly documented.
Source refers to interdependencies or essential-service continuity	Do not assign compound or cascading disruption without an explicit concurrent interaction or causal chain of secondary failures.

9. Independent coding limitation

No independent duplicate screening or coding was completed before submission. The original screening, extraction and coding were undertaken by one researcher, and no agreement statistic is reported.

10. Framework anchors

The codebook was derived by cross-mapping the following framework families. These sources anchor domain coverage; they are not themselves scored as study outcomes:

- WHO Health Emergency Preparedness, Response and Resilience (HEPR) architecture.
- WHO Framework for Health Emergency Preparedness and Response Capabilities for National Public Health Agencies (2026).
- ECDC Public Health Emergency Preparedness Assessment (PHEPA) capacities and first-cycle assessment approach.
- EU Preparedness Union Strategy and related EU health-crisis preparedness instruments.
- NATO 2026 Resilience Baseline Requirements, used only as an external benchmark for civil–military, mass-casualty and disruptive-health-crisis dimensions.
